# Opportunistic Treatment of Hepatitis C Infection Among Hospitalized People Who Inject Drugs (OPPORTUNI-C): A Stepped Wedge Cluster Randomized Trial

**DOI:** 10.1093/cid/ciad711

**Published:** 2023-11-22

**Authors:** Håvard Midgard, Kristian Braathen Malme, Charlotte Meinich Pihl, Riikka Mari Berg-Pedersen, Lars Tanum, Ingvild Klundby, Anne Haug, Ida Tveter, Ronny Bjørnestad, Inge Christoffer Olsen, Ane-Kristine Finbråten, Olav Dalgard

**Affiliations:** Department of Gastroenterology, Oslo University Hospital, Oslo, Norway; Department of Infectious Diseases, Akershus University Hospital, Lørenskog, Norway; Department of Infectious Diseases, Akershus University Hospital, Lørenskog, Norway; Institute of Clinical Medicine, University of Oslo, Oslo, Norway; Department of Medicine, Lovisenberg Diaconal Hospital, Oslo, Norway; Unger-Vetlesen Institute, Lovisenberg Diaconal Hospital, Oslo, Norway; Department of Addiction Medicine, Oslo University Hospital, Oslo, Norway; Department for Research and Development in Mental Health, Akershus University Hospital, Lørenskog, Norway; Faculty of Health Sciences, Oslo Metropolitan University, Oslo, Norway; Department of Microbiology, Oslo University Hospital, Oslo, Norway; Department of Acute Medicine, Oslo University Hospital, Oslo, Norway; Department of Infectious Diseases, Oslo University Hospital, Oslo, Norway; ProLAR Nett, Søgne, Norway; Department of Research Support for Clinical Trials, Oslo University Hospital, Oslo, Norway; Department of Medicine, Lovisenberg Diaconal Hospital, Oslo, Norway; Unger-Vetlesen Institute, Lovisenberg Diaconal Hospital, Oslo, Norway; Department of Infectious Diseases, Akershus University Hospital, Lørenskog, Norway; Institute of Clinical Medicine, University of Oslo, Oslo, Norway

**Keywords:** hepatitis C virus, people who inject drugs, model of care, pragmatic clinical trial, stepped wedge cluster randomized trial

## Abstract

**Background:**

We aimed to evaluate the efficacy of opportunistic treatment of hepatitis C virus (HCV) infection among hospitalized people who inject drugs (PWID).

**Methods:**

We performed a pragmatic, stepped wedge cluster randomized trial recruiting HCV RNA positive individuals admitted for inpatient care in departments of internal medicine, addiction medicine, and psychiatry at three hospitals in Oslo, Norway. Seven departments were sequentially randomized to change from control conditions (standard of care referral to outpatient care) to intervention conditions (immediate treatment initiation). The primary outcome was treatment completion, defined as dispensing the final package of the prescribed treatment within six months after enrolment.

**Results:**

A total of 200 HCV RNA positive individuals were enrolled between 1 October 2019 and 31 December 2021 (mean age 47.4 years, 72.5% male, 60.5% injected past 3 months, 20.4% cirrhosis). Treatment completion was accomplished by 67 of 98 (68.4% [95% confidence interval {CI}: 58.2–77.4]) during intervention conditions and by 36 of 102 (35.3% [95% CI: 26.1–45.4]) during control conditions (risk difference 33.1% [95% CI: 20.0–46.2]; risk ratio 1.9 [95% CI: 1.4–2.6]). The intervention was superior in terms of treatment completion (adjusted odds ratio [aOR] 4.8 [95% CI: 1.8–12.8]; *P* = .002) and time to treatment initiation (adjusted hazard ratio [aHR] 4.0 [95% CI: 2.5–6.3]; *P* < .001). Sustained virologic response was documented in 60 of 98 (61.2% [95% CI: 50.8–70.9]) during intervention and in 66 of 102 (64.7% [95% CI: 54.6–73.9]) during control conditions.

**Conclusions:**

An opportunistic test-and-treat approach to HCV infection was superior to standard of care among hospitalized PWID. The model of care should be considered for broader implementation.

**Clinical Trials Registration.** NCT04220645

## Teaser

Opportunistic HCV treatment among hospitalized people who inject drugs was superior to a referral-based standard of care in terms of treatment initiation and completion. The intervention was effective in all subgroups. Mortality was driven by liver cancer and renal failure.

## Graphical Abstract

**Graphical Abstract:**
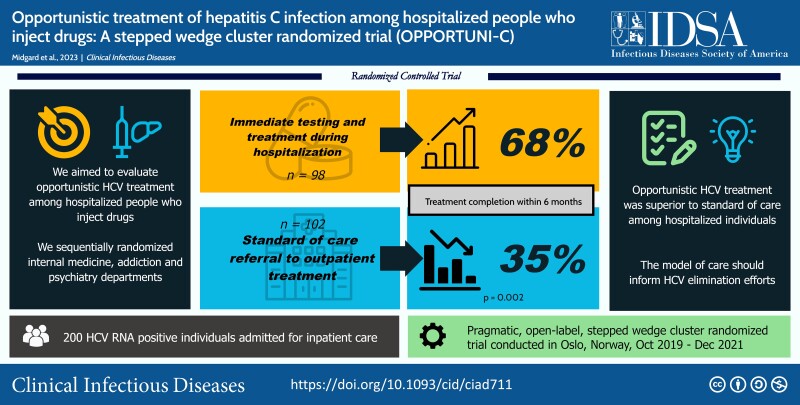
This graphical abstract is also available at Tidbit: https://tidbitapp.io/tidbits/opportunistic-treatment-of-hepatitis-c-infection-among-hospitalized-people-who-inject-drugs-opportuni-c-a-stepped-wedge-cluster-randomized-trial

**(See the Editorial Commentary by Rowan and Wyles on pages 591–3.)**

In Western Europe and North America 65%–80% of the hepatitis C virus (HCV) disease burden is attributable to injecting drug use [1]. People who inject drugs (PWID) therefore represent a priority population for testing and treatment to reach the World Health Organization's goal of eliminating HCV infection as a major public health threat within 2030 [2].

Despite direct-acting antiviral (DAA) therapy being safe and effective among PWID [3–9], treatment uptake in this population remains suboptimal [10, 11]. One of the critical obstacles to HCV care among PWID is the lack of treatment models adapted to marginalized individuals [12]. The current standard of care, involving referral of patients to specialist care at hospital outpatient clinics, is of limited value due to lack of retention in care [13, 14].

Although PWID are at high risk of hospitalization for skin and soft tissue infections and other drug-related harms [15, 16], hospital admissions are not sufficiently utilized for HCV testing and treatment [17]. An almost 4-fold increased risk of all-cause hospitalization has been shown among people with HCV infection in the United States [18], further highlighting the potential role of hospitalization as a venue for HCV treatment. Except for one small observational study reporting 66% treatment uptake among eligible inpatients in Australia [19] and one qualitative study addressing the concept [20], no study has assessed opportunistic HCV treatment among hospitalized individuals.

OPPORTUNI-C aimed to evaluate the efficacy of immediate testing and treatment of HCV infection among PWID admitted for inpatient care in internal medicine, addiction medicine, and psychiatry departments. To evaluate this intervention at the level of health service delivery, we used a stepped wedge cluster randomized trial design [21]. This design was chosen to facilitate a gradual and “naturalistic” implementation and to avoid contamination of the intervention and disappointment effects in unexposed clusters. We hypothesized that hospitalizations represent opportunities to engage PWID in HCV care more effectively than a referral-based standard of care.

## METHODS

### Study Design

OPPORTUNI-C was a pragmatic, open-label, multicenter, stepped wedge cluster randomized trial. The design involved a sequential rollout of the intervention over 8 time periods. Seven departments (clusters) of internal medicine (n = 3), addiction medicine (n = 2), and psychiatry (n = 2) at 3 hospitals in Oslo, Norway, were assigned to change from control (standard of care) to intervention conditions in a random order until all clusters were exposed to the intervention (Supplementary Figure 1). The study protocol has been published previously [22] and a full methods description is available in the Supplementary materials and the Statistical Analysis Plan. The study was registered with ClinicalTrials.gov (NCT04220645) on 1 October 2019.

The trial commenced on 1 October 2019, and the planned duration of each period was 2 months. Enrollment was affected by the coronavirus disease 2019 (COVID-19) pandemic, and the trial was temporarily stopped for 1 month during the first Norwegian lockdown in April 2020. As subsequent enrollment was almost halved, we increased the duration of the remaining 5 periods to reach the recruitment target.

The study was approved by the Regional Committee for Medical Research Ethics in Norway on 3 March 2019 (reference number 2019–128). The study was conducted according to the Declaration of Helsinki and International Conference on Harmonization Good Clinical Practice guideline. Written, informed consent was obtained from all participants.

### Participants

Participant inclusion criteria were (1) age > 18 years, (2) current HCV infection, defined as detectable HCV RNA, (3) admitted for inpatient care in one of the clusters, and (4) able to provide informed written consent. Participants were ineligible only if they (1) had ongoing HCV treatment, (2) were pregnant or breastfeeding, or (3) did not provide or withdrew their consent.

Screening for HCV infection was done according to usual practice and as soon as possible after admission. Following identification of any HCV RNA positive individual, the local microbiology department alerted a local investigator who obtained informed consent and facilitated enrollment in cooperation with the clinical staff.

### Randomization

Allocation was computer-generated and stratified according to expected cluster size (small, medium, large) to keep high HCV prevalence clusters separated regarding the timing of the intervention. The sequences were prepared by a statistician not involved in enrollment and kept in closed opaque envelopes. Concealment of a new step in the sequence was made available to the researchers on the day of transition and immediately disclosed to the clinical staff at the relevant cluster.

### Procedures

During intervention conditions, all participants were offered immediate HCV assessment and treatment initiation during hospitalization or as soon as possible after discharge. The intervention was delivered by the local investigator in cooperation with the responsible inpatient physician. Briefly, it comprised the following components: (1) *Liver disease staging* based on transient elastography or FIB-4 index; (2) *Pre-treatment counseling* at the discretion of the treating physician; (3) *DAA treatment initiation* following Norwegian HCV treatment recommendations, typically with oral fixed-dose pan-genotypic combinations sofosbuvir/velpatasvir for 12 weeks or glecaprevir/pibrentasvir for 8 weeks; (4) *Individualized follow-up* at the discretion of the treating physician, with support from the local low-threshold HCV clinic [9] or other facilities, as needed.

During control conditions, all enrolled participants were referred for outpatient HCV care following discharge in accordance with the established standard of care for hospitalized individuals.

Participants did not complete a conventional case report form, but key background variables were summarized in a standardized inclusion template in the electronic patient files at enrollment.

### Outcomes

The primary outcome was *treatment completion*, defined as dispensing the final 4-week package of the prescribed DAAs from the pharmacy within 6 months after enrollment. Failure to accomplish the primary outcome was noted either if no treatment had been dispensed (ie, loss to follow-up or other reasons), if treatment had been dispensed but completed later than six months after enrolment (ie, delayed treatment), or if the final package had not been dispensed (ie, treatment discontinuation).

Secondary outcomes were *treatment initiation* and *sustained virologic response (SVR).* Treatment initiation was defined as dispensing the first package of DAAs within 6 months after enrolment. SVR was defined as undetectable HCV RNA at least 4 weeks after the estimated date of end of treatment (SVR ≥ 4). Failure to achieve SVR was noted either if HCV RNA was detectable following end of treatment (ie, virologic failure), if no samples were available for SVR assessment (ie, loss to follow-up), or if no DAAs were dispensed (ie, no treatment).

Data on treatment completion and treatment initiation were extracted retrospectively by review of the “core medical record” in the electronic patient files 6 months after enrolment of the final participant. This record contains complete prescription and dispensation data from pharmacies nationwide within the previous 3 years. Data on SVR, baseline variables, and causes of death were obtained by retrospective review of the electronic hospital files and microbiology files from local and collaborating laboratories. No measures of adherence or records of protocol deviations were recorded.

### Statistical Methods

To show a 30% difference in effect size (60% intervention vs 30% control) for the primary outcome, with 85% power and 5% significance level, assuming a large intra-cluster correlation coefficient of 0.2, we planned to recruit on average 4 participants per cluster per period for a total of 224 participants (Supplementary Table 1).

Trial data are reported as mean (SD), median (interquartile range), or N (%) as appropriate. Data analysis followed an intention-to-treat principle according to cluster allocation regardless of what occurred, with no account of protocol non-adherence.

Outcomes are reported as proportions, risk differences, and risk ratios with 95% exact confidence intervals (CI). We analyzed treatment completion using mixed-effects logistic regression adjusted for calendar time with cluster as random effect, according to the Hussey and Hughes model [23]. We analyzed treatment initiation using Cox regression adjusted for calendar time with cluster as a shared frailty factor [24]. Time at risk for each participant was from the date of enrolment until the date of treatment, death, or 6 months after enrollment, whatever came first. Effect estimates are reported as adjusted odds ratios (aOR) or adjusted hazard ratios (aHR), and superiority of the intervention is claimed if a 2-sided *P*- value under the null hypothesis is < .05 in favor of the intervention.

We performed subgroup analyses using intervention × subgroup interaction according to pre-specified variables and post hoc analyses of mortality using Cox regression. As robustness analyses, we did a permutation test for the primary outcome with 10 000 random permutations of the cluster allocation [25] and analyzed the secondary outcome using a clustered sandwich estimator [26].

All analyses were performed using STATA 17 (College Station, Texas, USA) with the sample size calculation using the *steppedwedge* package [27].

## RESULTS

**Figure 1.**
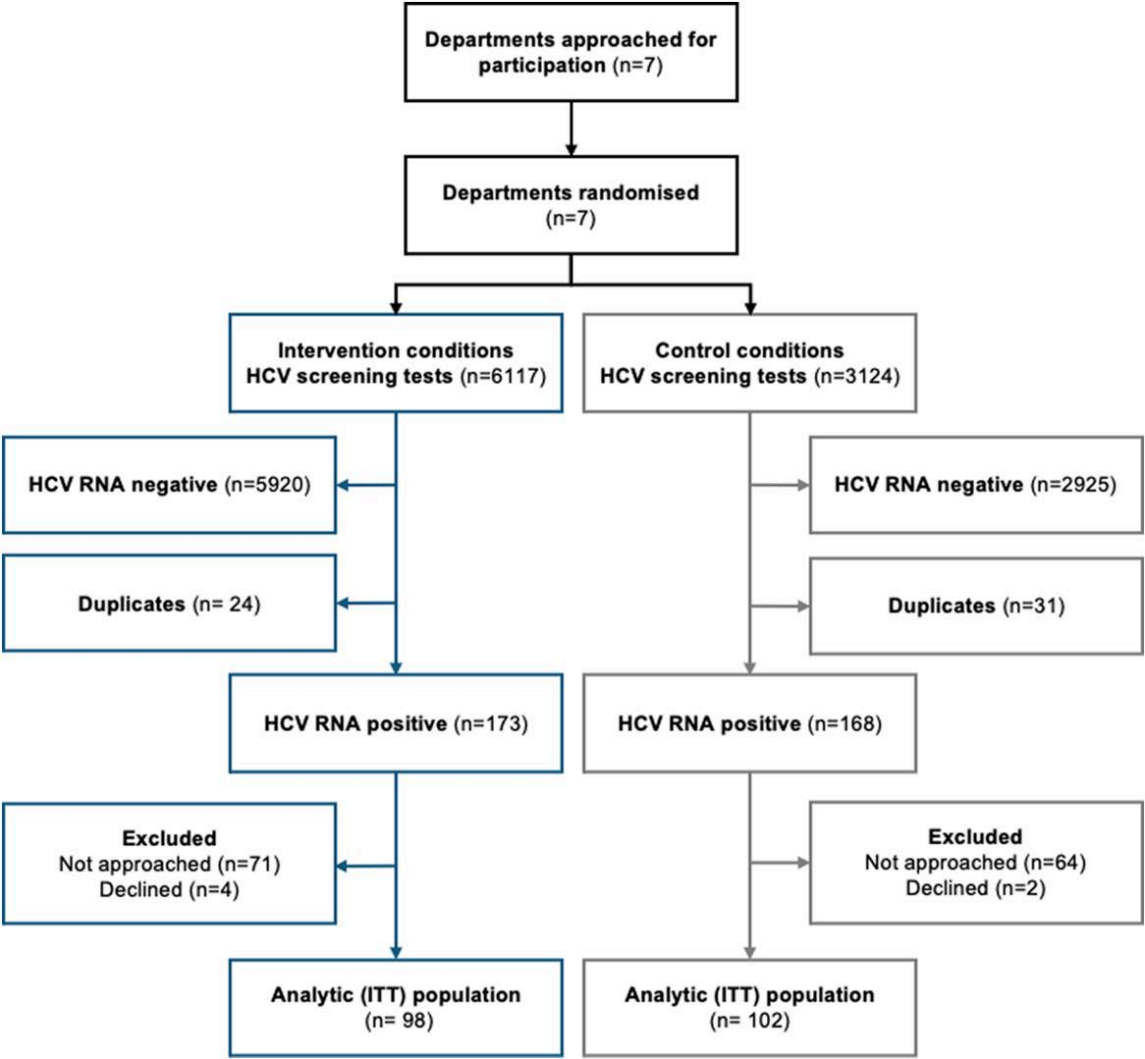
Flow of clusters and participants in OPPORTUNI-C by intervention condition. Abbreviations: HCV, hepatitis C virus; ITT, intention to treat.

Between 1 October 2019 and 31 December 2021, 200 individuals were enrolled in clusters of internal medicine (n = 107), addiction medicine (n = 65), and psychiatry (n = 28). A total of 9241 HCV screening tests were performed over the 8 time periods of the trial (Supplementary Table 2). Of 341 HCV RNA positive individuals identified, 141 (41.3%) were not included; 135 were not approached due to early discharge before potential enrolment, 6 were not willing to participate, whereas none were ineligible due to ongoing HCV treatment or pregnancy/breastfeeding (Figure 1). Despite stable screening activity, viremic rates and enrollment rates declined during the trial (Supplementary Figures 2 and 3).

Baseline characteristics were similar during intervention and control conditions (Table 1) and throughout the trial (Supplementary Table 3). The mean age was 47.4 years, the majority were male, and most had injected drugs in the previous 3 months. Liver cirrhosis was diagnosed in one-fifth of participants, and hepatocellular carcinoma was detected in 9 participants. Individuals enrolled in internal medicine were older and had more comorbidities than those enrolled in addiction medicine and psychiatry (Supplementary Table 4). The spectrum of discharge diagnoses is shown in Supplementary Table 5.

**Table 1.** Baseline Characteristics Summarized by Total and Intervention Condition

Variable	Total (n = 200)	Intervention (n = 98)	Control (n = 102)
Age, mean (SD)	47.4 (12.7)	48.0 (13.0)	46.8 (12.5)
Age groups			
20–29	21 (10.5)	11 (11.2)	10 (9.8)
30–39	40 (20.0)	16 (16.3)	24 (23.5)
40–49	51 (25.5)	21 (21.4)	30 (29.4)
50–59	52 (26.0)	33 (33.7)	19 (18.6)
60–80	36 (18.0)	17 (17.4)	19 (18.6)
Sex			
Male	145 (72.5)	69 (70.4)	76 (74.5)
Female	55 (27.5)	29 (29.6)	26 (25.5)
Housing status			
Rented/owned accommodation	124 (62.0)	64 (65.3)	60 (58.8)
Drug rehabilitation institution	10 (5.0)	7 (7.1)	3 (2.9)
Low-threshold institution	28 (14.0)	10 (10.2)	18 (17.7)
Prison	1 (0.5)	0 (0)	1 (1.0)
Homeless/on the street	37 (18.5)	17 (17.4)	20 (19.6)
Source of income^a^			
Part- or full-time job	26 (13.1)	14 (14.3)	12 (11.9)
Welfare pension	116 (58.6)	61 (62.9)	55 (54.5)
Social	52 (26.3)	20 (20.6)	32 (31.7)
Other	4 (2.0)	2 (2.1)	2 (2.0)
History of injecting drug use			
Yes	183 (91.5)	86 (87.8)	97 (95.1)
No	17 (8.5)	12 (12.2)	5 (4.9)
Recent (past 3 m) injecting drug use			
Yes	121 (60.5)	58 (59.2)	63 (61.8)
No	79 (39.5)	40 (40.8)	39 (38.2)
Recent sharing of injecting equipment^b^			
Yes	34 (28.1)	13 (22.4)	21 (33.3)
No	54 (44.6)	30 (51.7)	24 (38.1)
Unknown	33 (27.3)	15 (24.9)	18 (28.6)
Preferred injected drug^c^			
Heroin	114 (64.8)	54 (64.3)	60 (65.2)
Amphetamines	51 (29.0)	25 (29.8)	26 (28.3)
Other/mixed	11 (6.3)	5 (6.0)	6 (6.5)
Current opioid agonist therapy			
Yes	90 (45.0)	38 (38.8)	52 (51.0)
No	110 (55.0)	60 (61.2)	50 (49.0)
Opioid agonist therapy drug^d^			
Methadone	48 (53.3)	21 (55.3)	27 (51.9)
Buprenorphine	36 (40.0)	14 (36.8)	22 (42.3)
Buprenorphine-naloxone	2 (2.2)	0 (0)	2 (3.9)
Other	4 (4.4)	3 (7.9)	1 (1.9)
Stage of liver disease^e^			
Mild or no liver fibrosis	102 (52.0)	51 (52.0)	51 (52.0)
Intermediate fibrosis	54 (27.6)	25 (25.5)	29 (29.6)
Compensated cirrhosis	21 (10.7)	14 (14.3)	7 (7.1)
Decompensated cirrhosis	19 (9.7)	8 (8.2)	11 (11.2)
FIB-4 index, mean (SD)^f^	2.72 (5.95)	2.85 (7.69)	2.60 (3.43)
Hepatocellular carcinoma			
Yes	9 (4.5)	5 (5.1)	4 (3.9)
No or not assessed	191 (95.5)	93 (94.9)	98 (96.1)
Renal function			
eGFR >60 mL/min/1.73 m^2^	188 (94.0)	89 (90.8)	99 (97.1)
eGFR 30–59 mL/min/1.73 m^2^	8 (4.0)	6 (6.1)	2 (2.0)
eGFR <30 mL/min/1.73 m^2^	4 (2.0)	3 (3.1)	1 (1.0)
HIV coinfection			
Yes	6 (3.0)	3 (3.1)	3 (2.9)
No	169 (84.5)	86 (87.8)	83 (81.4)
Not assessed	25 (12.5)	9 (9.2)	16 (15.7)
HBV coinfection (HBsAg+)			
Yes	1 (0.5)	1 (1.0)	0 (0)
No	180 (90.0)	93 (94.9)	87 (85.3)
Not assessed	19 (9.5)	4 (4.1)	15 (14.7)
HCV genotype			
Genotype 1	47 (23.5)	16 (16.3)	31 (30.4)
Genotype 2	7 (3.5)	1 (1.0)	6 (5.9)
Genotype 3	48 (24.0)	20 (20.4)	28 (27.5)
Genotype 4–6	6 (3.0)	2 (2.0)	4 (3.9)
Not genotyped	92 (46.0)	59 (60.2)	33 (32.4)
Days of hospitalization, median (IQR)	6 (3–13)	5 (2–13)	7 (4–13)
Main discharge diagnosis			
Drug related	93 (46.5)	39 (39.8)	54 (52.9)
Infectious diseases	33 (16.5)	22 (22.5)	11 (10.8)
Gastroenterology/hepatology	24 (12.0)	10 (10.2)	14 (13.7)
Mental health	15 (7.5)	9 (9.2)	6 (5.9)
Cardiopulmonary	14 (7.0)	8 (8.2)	6 (5.9)
Alcohol related	7 (3.5)	4 (4.1)	3 (2.9)
Other	14 (7.0)	6 (6.1)	8 (7.8)
Charlson comorbidity index			
0–1	101 (50.5)	41 (41.8)	60 (58.2)
2–3	43 (21.5)	30 (30.6)	13 (12.8)
4–5	33 (16.5)	16 (16.3)	17 (16.7)
≥6	23 (11.5)	11 (11.2)	12 (11.8)
Charlson comorbidity index, mean (SD)	2.6 (2.3)	2.8 (2.4)	2.5 (2.2)

Numbers are shown as n (%) unless otherwise indicated. Missing values are excluded from percentages.

Abbreviations: FIB-4, fibrosis-4; GFR, glomerular filtration rate; HBV, hepatitis B virus; HIV, human immunodeficiency virus; IQR, interquartile range; SD, standard deviation.

^a^Missing data for 2 participants (1 intervention, 1 control).

^b^Among those with recent (past 3 m) injecting drug use.

^c^Among those with a history of injecting drug use; missing data for 7 participants (2 intervention, 5 control).

^d^Among those with current opioid agonist therapy.

^e^Based on liver stiffness measurements in 86, FIB-4 index in 107, and imaging in 3 participants; missing data for 4 control participants.

^f^Among 193 participants with an available FIB-4 index.

Treatment completion within 6 months (Table 2) was accomplished by 67 of 98 (68.4% [95% CI: 58.2–77.4]) during intervention conditions and by 36 of 102 (35.3% [95% CI: 26.1–45.4]) during control conditions (risk difference 33.1% [95% CI: 20.0–46.1]; risk ratio 1.9 [95% CI: 1.4–2.6]). In mixed-effects logistic regression, the intervention was superior to standard of care (aOR 4.8 [95% CI: 1.8–12.8]; *P* = .002) with no significant effect of secular trends (aOR 1.0 [95% CI: .95–1.06]). Cluster effects were moderate (SD 0.16 [95% CI: .01–1.69]) with an intra-cluster correlation coefficient of 0.046 (95% CI: .005–.339). The permutation test confirmed the result (*P* = .008).

**Table 2.** Overview of Primary and Secondary Outcomes Summarized by Total and Intervention Condition

	Total (n = 200)	Intervention (n = 98)	Control (n = 102)	Risk Difference	Risk Ratio (95% CI)
*Primary outcome*					
Treatment completion within 6 months					
N	103	67	36		
% (95% CI)	51.5 (44.3–58.6)	68.4 (58.2–77.4)	35.3 (26.1–45.4)	33.1 (20.0–46.1)	1.9 (1.4–2.6)
Failure to accomplish completion, n (%)					
Delayed treatment initiation	39 (19.5)	7 (7.1)	32 (31.4)	NA	NA
Treatment discontinuation	13 (6.5)	10 (10.2)	3 (2.9)		
Short life expectancy or death	15 (7.5)	6 (6.1)	9 (8.8)		
Declined treatment	2 (1.0)	1 (1.0)	1 (1.0)		
Prolonged treatment	1 (0.5)	1 (1.0)	0 (0)		
Loss to follow-up	27 (13.5)	6 (6.1)	21 (20.6)		
*Secondary outcomes*					
Treatment initiation within 6 months					
n	131	84	47		
% (95% CI)	65.5 (58.5–72.1)	85.7 (77.2–92.0)	46.1 (36.2–56.2)	39.6 (27.7–51.5)	1.9 (1.5–2.3)
Treatment initiation within data lock					
n	159	87	72		
% (95% CI)	79.5 (73.2–84.9)	88.8 (80.8–94.3)	70.6 (60.7–79.2)	18.2 (7.4–29.0)	1.3 (1.1–1.5)
SVR ≥4					
n	126	60	66		
% (95% CI)	63.0 (55.9–69.7)	61.2 (50.8–70.9)	64.7 (54.6–73.9)	−3.5 (−16.9–9.9)	0.95 (.76–1.2)
SVR ≥12					
n	104	51	53		
% (95% CI)	52.0 (44.8–59.1)	52.0 (41.7–62.2)	52.0 (41.8–62.0)	0.0 (−0.14–0.14)	1.00 (.77–1.3)
Failure to accomplish SVR ≥4, n (%)					
Virologic failure	8 (4.0)	6 (6.1)	2 (2.0)	NA	NA
Loss to follow-up (missing SVR data)	25 (12.5)	21 (21.4)	4 (3.9)		
No treatment	41 (20.5)	11 (11.2)	30 (29.4)		

Abbreviations: CI, confidence interval; NA, not applicable; SVR, sustained virologic response.

**Figure 2.**
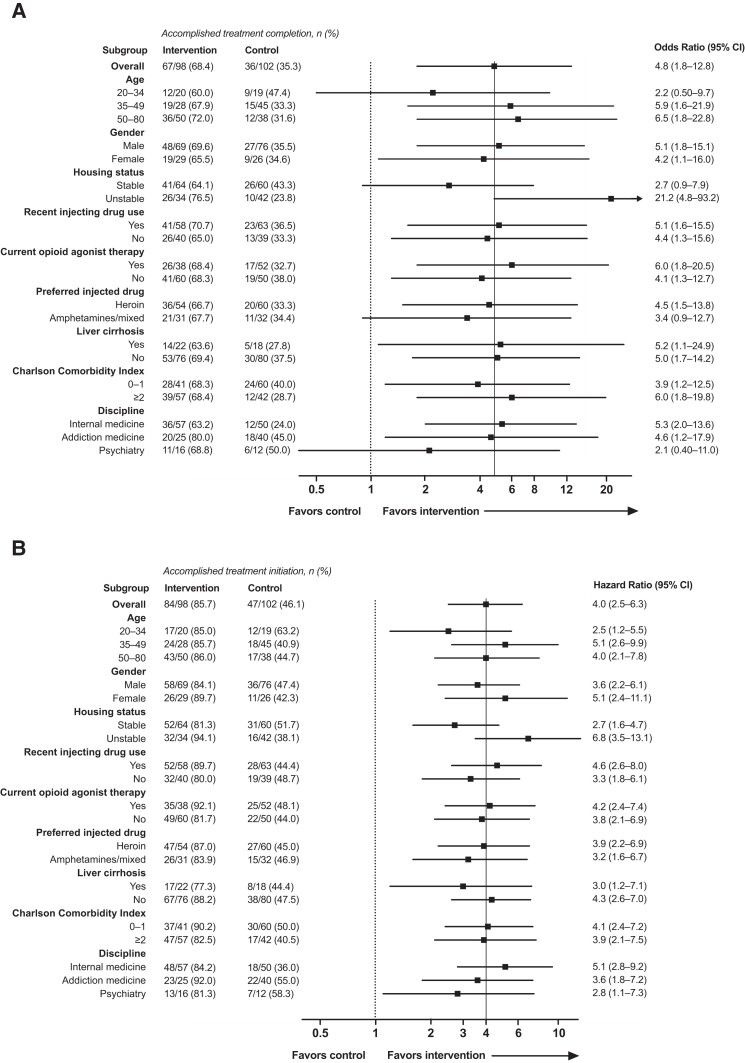
Subgroup analyses of (*A*) the primary outcome (treatment completion within 6 months) and (*B*) the secondary outcome (treatment initiation within 6 months). Abbreviation: CI, confidence interval.

Retention in care at 6 months was higher during intervention than during control conditions but the difference was less prominent at data lock (Supplementary Figure 4). Most received pan-genotypic treatment with sofosbuvir/velpatasvir (55.3%) or glecaprevir/pibrentasvir (26.2%), and one-half of treatments during intervention conditions were self-administered without aid from other services (Supplementary Table 6). Subgroup analysis (Figure 2*A*) indicated a homogeneous intervention effect but with a signal that the intervention had a stronger impact among those with unstable housing (interaction *P* = .004).

**Figure 3.**
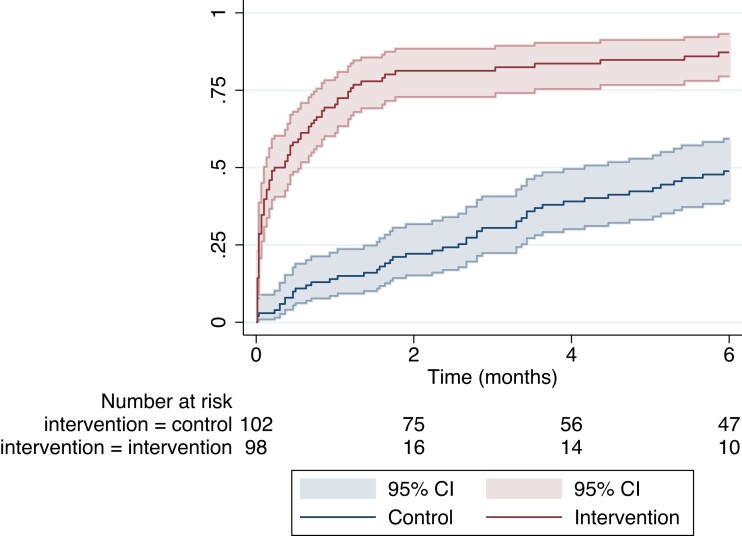
Kaplan–Meier plot of time to treatment initiation. Red lines represent the proportion of participants in intervention conditions and blue lines represent the proportion of participants in control conditions. Shaded areas indicate 95% confidence intervals.

Treatment initiation within 6 months (Table 2; Supplementary Table 6) was accomplished by 84 of 98 (85.7% [95% CI: 77.2–92.0]) during intervention conditions and by 47 of 102 (46.1% [95% CI: 36.2–56.2]), during control conditions (risk difference 39.6% [95% CI: 27.7–51.5]; risk ratio 1.9 [95% CI: 1.5–2.3]). The hazard of treatment (Figure 3) was significantly higher during intervention compared to control conditions (aHR 4.0 [95% CI: 2.5–6.3]; *P* < .001) with no significant effect of secular trends (aHR 1.0; 95% CI: .98–1.03). Robustness analysis confirmed the result (aHR 4.0 [95% CI: 2.4–6.5]; *P* < .001). The intervention effect was homogeneous across subgroups (Figure 2*B*).

SVR ≥4 (Table 2) was documented in 60 of 98 (61.2% [95% CI: 50.8–70.9]) during intervention conditions and in 66 of 102 (64.7% [95% CI: 54.6–73.9]) during control conditions (risk difference −3.5% [95% CI: −16.9–9.9]; risk ratio 0.95 [95% CI: .76–1.2]). Failure to achieve SVR was largely explained by loss to follow-up (ie, missing data) during intervention and by lack of treatment during control conditions (Table 2). Among 141 participants with complete dispensation within data lock, 120 (85.1%) achieved SVR ≥4 (Supplementary Table 7).

Post hoc analysis of mortality (Supplementary Table 8) showed that 24 participants (12.0%) died during the study period. The main causes of death were liver-related (n = 7) or due to end-stage renal disease (n = 6), and no deaths were related to HCV treatment. Of 9 participants with hepatocellular carcinoma, 6 were advanced cases who died within 2 months after enrollment. There was a trend of higher mortality during intervention (11.5/100 PY [95% CI: 6.3–19.2]) compared to control conditions (4.8/100 PY [95% CI: 2.3–8.9]), but Cox regression showed no significant effect of the intervention (aHR 2.0; 95% CI: .7–5.5; *P* = .19) and no significant effect of secular trends (aHR 1.0; 95% CI: .93–1.1; *P* = .89).

## DISCUSSION

Opportunistic HCV treatment among hospitalized PWID was superior to a referral-based standard of care in terms of treatment completion and treatment initiation. The results could change clinical practice and health policy internationally and should inform HCV elimination efforts among PWID.

Treatment efficacy was lower than in previous studies of HCV treatment among PWID [3–9, 28]. Our results are more in line with a recent trial from the United States, reporting rates of treatment initiation, completion, and SVR of 83%, 68%, and 61% in intention-to-treat analysis [29]. This probably reflects the pragmatic features of both trials, enabling recruitment of more marginalized individuals than in previous studies. In the present study, failure to accomplish the primary outcome was largely explained by loss to follow-up and delayed treatments, and consistent with literature [3], rates of treatment discontinuation were low. Although the benefit of early treatment was limited by relatively low SVR, it could be explained by a higher proportion of missing data during intervention conditions.

The superiority of the intervention was driven by a considerably shorter time to treatment during intervention conditions. This is of clinical and public health significance because persisting viraemia can lead to onward HCV transmission among those with ongoing risk behaviors. Although treatment uptake was slower during control conditions, it increased after the protocol-specified 6 months follow-up and toward data lock. We observed that these individuals had often been engaged in low-threshold HCV treatment in the City of Oslo and had not received specialist care at the hospital outpatient clinics as planned. The intervention effect may therefore be underestimated compared to settings without access to similar low-threshold care.

Subgroup analysis also favored the intervention among the most marginalized individuals. Notably, the intervention seemed more effective among those with unstable housing. For individuals at risk of loss to outpatient follow-up due to homelessness (ie, lack of contact address), the intervention may have enabled retention in care by linkage to low-threshold facilities.

Despite stable screening rates, viremic rates declined in all clusters during the trial, particularly following the COVID-19 pandemic. Due to the natural dominance of intervention observations arising from later calendar times inherent to the stepped wedge design, the viremic prevalence was 50% lower during intervention conditions than during control conditions. Although the declining rates could be attributed to the pandemic, it may also reflect the marked decline in HCV RNA prevalence reported among PWID in Oslo [30].

Mortality was higher than reported in previous HCV treatment studies among PWID [3], which largely have comprised of community dwelling individuals recruited from the outpatient setting. Mortality was mainly driven by underlying chronic diseases, reflecting recruitment of an acutely hospitalized and ageing population with a high prevalence of advanced liver disease and renal disease. The potential trend in increased mortality during intervention conditions could be explained by the small sample and 3 cases of suicide in the intervention group but also due to recruitment of an increasingly marginalized population in a period where Norway was approaching HCV elimination.

To our knowledge, this is the first controlled study to evaluate an opportunistic HCV treatment model among hospitalized individuals. Key strengths relate to the pragmatic features of the trial, including (1) broad recruitment of marginalized individuals, (2) the use of clinical infrastructures with minimal research-specific frameworks, (3) extraction of routinely collected data without the need for individual follow-up, and (4) an intention-to-treat principle for data analysis. Although the stepped wedge design is unconventional with numerous methodological complexities, it is a pragmatic design considered appropriate for evaluation of health delivery interventions with political, logistical, and statistical advantages over an individual-randomized or a parallel cluster randomized design [21].

Together, these features have ensured representativeness of the study population, eliminated the impact of loss to follow-up and generated optimal conditions for generalizability at a low cost. Taken a step further, the approach could serve as a model for addressing other health problems among PWID opportunistically. However, the intervention relied on the unrestricted access to DAAs across healthcare settings that is available in Norway. Implementation would be more difficult in countries where treatment access is restricted by health insurance authorization and in countries where hospital formularies may be restricted to selected medications.

The trial had several limitations. First, because recruitment was done with study personnel and participants knowing the treatment allocation, potentially influencing screening activity and participation, the trial is at risk of selection bias between the intervention conditions. Second, the stepped wedge design is associated with potential confounding with time, an effect that may have been augmented by the COVID-19 pandemic and subsequent trial prolongation. However, we found no evidence that underlying secular trends had influenced the intervention effect. Third, the estimated effect sizes are imprecise. This could have been improved by increasing the number of participating clusters instead of increasing cluster size. Finally, the primary outcome remains a proxy. However, our data largely validate registry-based proxies as a pragmatic correlate for cure in marginalized populations. Given that good results of DAA treatment have been shown with suboptimal adherence [6, 31] or treatment shortened to four weeks [32], we expect that high SVR rates have been achieved also among those with missing data. Thus, virologic cure is probably underestimated among intervention participants.

In conclusion, we provide evidence that opportunistic HCV treatment is superior to a referral-based standard of care among hospitalized individuals. Hospitalizations should be utilized for testing and treatment of HCV infection and the model of care could represent a key strategy moving forward in the global response to the HCV epidemic.

## Supplementary Data

Supplementary materials are available at *Clinical Infectious Diseases* online. Consisting of data provided by the authors to benefit the reader, the posted materials are not copyedited and are the sole responsibility of the authors, so questions or comments should be addressed to the corresponding author.

## Supplementary Material

ciad711_Supplementary_Data

## Notes

***Author Contributions.*** Conception and design: O. D., H. M., A.-K. F., I. C. O. Collection and assembly of data: H. M., O. D., A.-K. F., K. B. M., C. M. P., R. M., B.-P., A. H., I. T., I. K. Analysis and interpretation of data: H. M., O. D., A.-K. F., K. B. M., R. B. L. T., I. C. O.. Drafting of the article: H. M.. Critical revision for important intellectual content: H. M., O. D., A.-K. F., K. B. M., C. M. P., R. M. B.-P., A. H., I. T., L. T., I. K., R. B., I. C. O. Final approval of the article: H. M., O. D., A.-K. F., K. B. M., C. M. P., R. M. B.-P., A. H., I. T., L. T., I. K., R. B., I. C. O. Statistical expertise: I. C. O., H. M. Obtaining of funding: O. D., H. M., A.-K. F. Administrative, technical, or logistic support: A, U., H. R.. H. M., K. B. M., A.-K. F., and O. D. have directly accessed and verified the underlying data reported in the manuscript and take responsibility for the integrity of the data and the accuracy of the data analysis. All authors had access to all the data and accept the responsibility to submit for publication. No compensation was provided to the authors. An earlier version of this manuscript was posted as a preprint on 8 December 2022 on the following SSRN sever: https://ssrn.com/abstract=4293865 or http://dx.doi.org/10.2139/ssrn.4293865

***Acknowledgments.*** The authors thank all study participants for their contribution to research and wishes to acknowledge the following members of the study group:

Protocol steering committee: O. D., H. M., A.-K. F., K. B. M., L. T., R. B., I. C. O.

User involvement: R. B., proLAR Nett, Søgne.

Akershus University Hospital: O. D., H. M., K. B. M., L. T., H. R., M. H., K. B., L. Z., D. E. H., T. L., F. R., S. K., T. H. F.

Oslo University Hospital: H. M., A. H., I. T., R. M. B.-P., I. K., . S.-J., I. C. O., S. L. M., H. E., L. L., D. N., B.-M. B., A. L.

Lovisenberg Diaconal Hospital: A.-K. F., C. M. P., H. L., H. A., I. G., L. R., H. M. N., A. N., A. T., J. V., A. U.

***Financial support*.** This work was supported by a research grant from the Southern and Eastern Norway Regional Health Authority (grant number 2020011). The funder of the study had no role in study design, data collection, data analysis, data interpretation, or writing of the report.
