# Supplementary material for: Opportunistic Treatment of Hepatitis C Infection Among Hospitalized People Who Inject Drugs (OPPORTUNI-C): A Stepped Wedge Cluster Randomized Trial
Source: Clin Infect Dis. 2023 Nov 22;78(3):582–90. doi: 10.1093/cid/ciad711 (PMC10954343; doi:10.1093/cid/ciad711)
Supplement: ciad711_Supplementary_Data [file ciad711_supplementary_data.zip › Statistical analysis plan v3.0.pdf]

1. Administrative information

Trial title: Opportunistic treatment of hepatitis C infection: A pragmatic stepped wedge cluster randomized trial of immediate treatment initiation among hospitalized people who inject drugs (OPPORTUNI-C)

Trial registration: ClinicalTrials.gov NCT04220645.
https://clinicaltrials.gov/ct2/show/NCT04220645?term=opportuni-c&draw=2&rank=1

SAP Version: 3.0 (27 March 2023)

Changes from previous version (v.2.1):
- specification of time frame for secondary outcome treatment initiation
- addition of protocol specified secondary outcome SVR
- specification of handling of missing data

Protocol version: Protocol published June 2020 (1).

Midgard, H., et al. (2020). "Opportunistic treatment of hepatitis C virus infection (OPPORTUNI-C): study protocol for a pragmatic stepped wedge cluster randomized trial of immediate versus outpatient treatment initiation among hospitalized people who inject drugs." Trials. 2020 Jun 15;21(1):524. doi: 10.1186/s13063-020-04434-8.

Trial status: The first study participant was enrolled on 1 October 2019. The final study participant was enrolled on 31. December 2021. A total of 210 participants were enrolled.

Roles and responsibility:
- Håvard Midgard, Department of Infectious Diseases, Akershus University Hospital and Oslo University Hospital. Norway. Coordinating and primary investigator. Responsible for data analysis, interpretation, and writing of the manuscript.
- Olav Dalgard, Department of Infectious Diseases, Akershus University Hospital, Norway. Project leader. Responsible for data interpretation and critical review of the manuscript.
- Ane-Kristine Finbråten, Department of Medicine, Lovisenberg Diaconal Hospital, Norway. Responsible for data interpretation and critical review of the manuscript.
- Kristian Braathen Malme, Department of Infectious Diseases, Akershus University Hospital, Norway. Responsible for data interpretation and critical review of the manuscript.
- Inge Christoffer Olsen, Department of Research Support for Clinical Trials, Oslo University Hospital, Norway. Responsible for data analysis supervision, interpretation, and critical review of the manuscript.

Signatures:

Håvard Midgard	Inge Christoffer Olsen	Olav Dalgard
SAP writer and PI	Senior Statistician	Project leader

2. Introduction

Background and rationale:
Hepatitis C virus (HCV) infection is a leading cause of liver cirrhosis and hepatocellular carcinoma (2, 3), and 58 million people are living with chronic HCV infection globally (4). In Western Europe, two-thirds of the HCV disease burden is attributable to injecting drug use (5). People who inject drugs (PWID) therefore represent a priority population for testing and treatment in order to reach the World Health Organization goal of eliminating HCV infection as a major public health threat within 2030 (6).

Direct-acting antiviral (DAA) therapy provide virologic cure in more than 95% after 8-12 weeks of oral treatment (7). Although clinical trials and cohort studies among PWID have demonstrated equally high efficacy (8-14), most studies are limited by selection biases and small sample sizes, not sufficiently reflecting that real-world HCV care may be complicated by suboptimal adherence (15), loss to follow-up (11), and reinfection following treatment (16). There is therefore a need for more generalizable data on successful models of care (17).

One of the critical obstacles to HCV care among PWID is the lack of treatment models within specialist health care adapted to marginalized individuals (18-20). The current standard of care, involving referral of patients to hospital outpatient clinics, is of limited value due to low attendance and lack of retention in the care cascade (21-24). Although PWID are at high risk of hospitalization for injection-related infectious diseases, drug-related complications and mental health disorders, hospital admissions are not utilized for HCV treatment initiation (25).

We hypothesized that hospitalizations represent opportunities to engage marginalized PWID in HCV care more effectively than the referral-based standard of care.

Objectives:
The primary objective of OPPORTUNI-C was to evaluate the efficacy of an “opportunistic” strategy with immediate HCV treatment among PWID admitted for emergency inpatient care in departments of internal medicine, addiction medicine, and psychiatry.

Secondary objectives not covered by this SAP include to calculate the incidence of HCV reinfection following treatment, to assess the frequency of resistance-associated substitutions, and to evaluate HCV testing uptake and HCV RNA prevalence before and during the trial.

3. Study methods

Trial design: OPPORTUNI-C was a multicentre, stepped wedge cluster randomized trial (26, 27) recruiting participants from departments of internal medicine (n=3), addiction medicine (n=2), and psychiatry (n=2) at Akershus University Hospital, Oslo University Hospital, and Lovisenberg Diaconal Hospital, Norway.

During control conditions, participants were referred for outpatient HCV care following discharge in accordance with the established standard of care for hospitalized individuals. During intervention conditions, participants were offered non-invasive liver disease assessment, immediate pan-genotypic DAA treatment initiation, and individualized follow-up.

Rationale for trial design: Cluster randomization at department-level instead of individual randomization was chosen to 1) evaluate the intervention effect at the level of health service

delivery and 2) eliminate the risk of “contamination” of the intervention between local health care providers or study participants. The stepped wedge design was chosen over a parallel cluster design because 1) a sequential rollout enabled a gradual implementation across heterogeneous centers; 2) assuming superiority, it was considered unreasonable to offer the intervention to only half of the clusters; and 3) it was considered a more statistically robust design due to an anticipated large intra-cluster correlation.

Pragmatic clinical trial approach: To mimic usual clinical practice as closely as possible and generate optimal conditions for generalizability at a low cost (28), we applied the following pragmatic clinical trial features: 1) recruitment from a routine clinical setting; 2) broad eligibility criteria; 2) minimal research-specific frameworks with use of existing clinical infrastructures; 3) flexible intervention delivery; 4) clinically relevant and easily available outcome measures; 5) extraction of routinely collected data without requirements for individual follow-up; and 6) data analysis according to an intention-to-treat principle.

Randomization and blinding: The departments were sequentially randomized to change from standard of care to intervention conditions between 1 October 2019 and 31 December 2021 (Table 1). Allocation was computer-generated and stratified according to cluster size to keep high prevalence clusters separated with regards to the timing of the intervention. Concealment of a new step in the allocation sequence was made available to the researchers on the day of transition and immediately disclosed to the clinical staff at the relevant department. This was a completely open trial, and all participants, study personnel and outcome assessors were unblinded to the intervention.

Sample size: Statistical power analysis was performed in STATA 15 using the *steppedwedge* command (29). To show a 30% difference in effect size (60% during intervention conditions vs. 30% in during control conditions) for the primary outcome, with 85% power and 5% significance level, assuming a large intra-cluster correlation coefficient of 0.2, we planned to recruit on average 4 participants per cluster per period for a total of 224 participants. A sensitivity analysis showing power and sample size estimates for a range of effect sizes are shown in Supplementary Table 1.

Assumptions of effects sizes and intra-cluster correlation were conservative and based on data from clinical databases at Akershus University Hospital (data on file) and a low-threshold HCV clinic in downtown Oslo (13). The stepped wedge design is considered appropriate and effective in situations with an expected large intra-cluster correlation, as is retains the statistical power available compared to alternative designs (30).

Framework: The null hypothesis that there is no effect of the intervention will be tested using the statistical methods described below. Superiority of the intervention over the control will be claimed if the p-value under the null hypothesis is less than 0.05.

Interim analysis and stopping guidance: There were no interim analysis or stopping guidelines, except what was part of usual practice if any severe adverse events occur.

Timing of outcome assessments and final analysis: All outcomes will be analysed collectively 6 months after enrolment of the final study participants. A detailed schedule of enrolment, interventions, assessments, and visits is included in the published protocol.

4. Statistical principles

Confidence intervals and significance level: The level of statistical significance is set at 0.05 (two-sided). 95% confidence intervals will be presented.

Adherence and protocol deviations: The primary analysis will follow an intention-to-treat principle according to cluster randomization status regardless of what occurred, with no account taken of protocol non-adherence or protocol deviances. No measures of adherence or records of protocol deviations have been collected during the trial.

Analytic population: The analytic population will be the intention-to-treat population defined as all participants fulfilling the eligibility criteria as defined in section 5 below.

5. Trial population

Eligibility: There were no strict pre-specified cluster eligibility criteria. Selection was based on clinical experience that individuals with HCV infection in the Oslo area are well represented in these departments, collectively covering a population of approximately one million.

Participant eligibility criteria were 1) age > 18 years; 2) current HCV infection, defined as detectable HCV RNA; 3) admitted for inpatient care; and 4) able to provide informed written consent. Participants were ineligible only if they had ongoing HCV treatment or withdrew their consent.

Recruitment: Information to be included in the CONSORT diagram are departments approached, departments randomized, participants screened, HCV RNA positive individuals, participants excluded, and eligible participants included in the analytic (intention-to-treat) population (Figure 1). Reasons for exclusion will be reported. Information to be included in the stepped-wedge recruitment diagram are the number of screened, viremic (HCV RNA positive), and enrolled (eligible) participants for each cluster period of the trial (Table 1). Inclusion rates and estimated HCV RNA prevalence for each cluster period will also be reported.

Withdrawal/Follow-up: Levels of study withdrawal will be reported if applicable. Levels of loss to follow-up are not relevant, because all outcome data will be evident from the patient files without the need for individual follow-up (see Outcome definitions).

Baseline patient characteristics: Characteristics at baseline for all study participants will be summarized by randomization group, cluster and total, and reported as N (%) or mean (SD) as appropriate according to Table 2. Treatment characteristics (time to treatment, treatment regimens and follow-up model) for participants who initiated treatment will be reported according to Table 4.

6. Analysis

Outcome definitions:

1) Primary outcome: Treatment completion (yes vs. no), defined as dispensing of the final four-week package of the prescribed DAAs within 6 months after enrolment.
2) Secondary outcomes:

a) Treatment initiation (yes vs. no), defined as dispensing of at least one package of DAAs within 6 months after enrolment.
b) Sustained virologic response (yes vs. no), defined as undetectable HCV RNA at least four weeks after the estimated date of end of treatment (SVR≥4).

Treatment completion and treatment initiation will be assessed by retrospective review of the "core medical record" in the electronic patient files 6 months after enrolment of the final participant (data lock). This record captures all prescriptions nationwide within the previous three years and include the name of the prescriber and the dates of prescription and dispensing of all prescribed drugs from the pharmacy. SVR will be assessed by retrospective review of the electronic hospital files and microbiology files from local and collaborating laboratories.

Analysis methods: Treatment completion will be analyzed as a binary variable using mixed effect logistic regression with intervention and calendar time as fixed effects and department as random effect (Stata code: *melogit completion intervention time || cluster:, or*) according to the Hussey and Hughes model (31). As robustness analysis, the same model will be analyzed using Generalized Estimating Equations (Stata code: *xtset cluster; xtlogit completion intervention time, pa*).

Treatment initiation will be analysed as time-to-event using Cox regression adjusted for calendar time with department as a shared frailty factor. The time at risk for each participant will be from the date of enrolment until the date of treatment, death, or six months after enrolment, whatever came first. As robustness analysis, the same model will be used without department as shared frailty factor, but where the variance is estimated using a clustered sandwich estimator. There will be no adjustment for other covariates in the primary analyses. The covariates in the Cox model will be tested for the proportional hazard′s assumption using Schoenfeld residuals and log–log transformation of the failure function. Kaplan Maier plots of time to treatment initiation will be performed (Figure 2).

All outcomes will be reported as proportions, risk differences, and risk ratios with 95% exact confidence intervals. Effect sizes form regression models will be presented as odds ratios or hazard ratios with 95% confidence intervals (Table 3). Random effects will be presented as the estimated standard deviation of the intercept on the logit scale. Model-based estimates for underlying temporal trends in the primary outcome for participants in control conditions and intervention conditions for each cluster-period will be calculated as predictive margins with 95% confidence intervals (Supplementary Figure 1).

Additional analyses: The cascade of care among all HCV RNA positive individuals will be shown at 6 months following enrolment and at data lock. The number of individuals with enrolment, prescription, initiation, and completion of treatment will be reported according to control and intervention conditions, respectively (Figure 3).

Subgroup analysis: Subgroup analyses of the primary outcome will be performed according to the following pre-specified background variables a-priory hypothesized to be associated with the outcome: age groups (20-35, 35-49, 50-80), gender (male vs female), housing status (stable vs. unstable), history of injecting drug use (yes vs. no), recent injecting drug use (yes vs. no), preferred injected drug (amphetamines vs. opioids), opioid agonist treatment (yes vs. no), liver cirrhosis (yes vs. no), Charlson Comorbidity Index (1, 2-3, ≥4) and discipline (internal medicine, addiction medicine, psychiatry). Subgroup point estimates and 95% confidence intervals will be presented as a forest plot and calculated using mixed effect logistic regression

with intervention x subgroup interaction and calendar time as fixed effects and department as random effect (Figure 4). A similar subgroup analysis for the secondary outcome treatment initiation will also be performed (Supplementary Figure 2, not shown here).

Missing data: Because outcome data will be collected from the "core medical record", there will be no missing data for the primary or secondary outcomes and no need for statistical methods to handle this. Missing SVR data will be handled using worst case imputation and assigned as failure. Missing data for baseline variables will be reported.

Harms: All severe adverse events occurring during the study period was followed up prospectively for each participant by the treating physician in accordance with usual clinical practice. DAA side effects are generally mild and transient and will not be collected nor reported. Causes of death for all individuals who died during the study period will be reported. High levels of somatic comorbidities with high competing death risks are to be expected.

Statistical software: All analyses will be performed using STATA 17 (College Station, TX, USA).

7. Proposed tables and figures

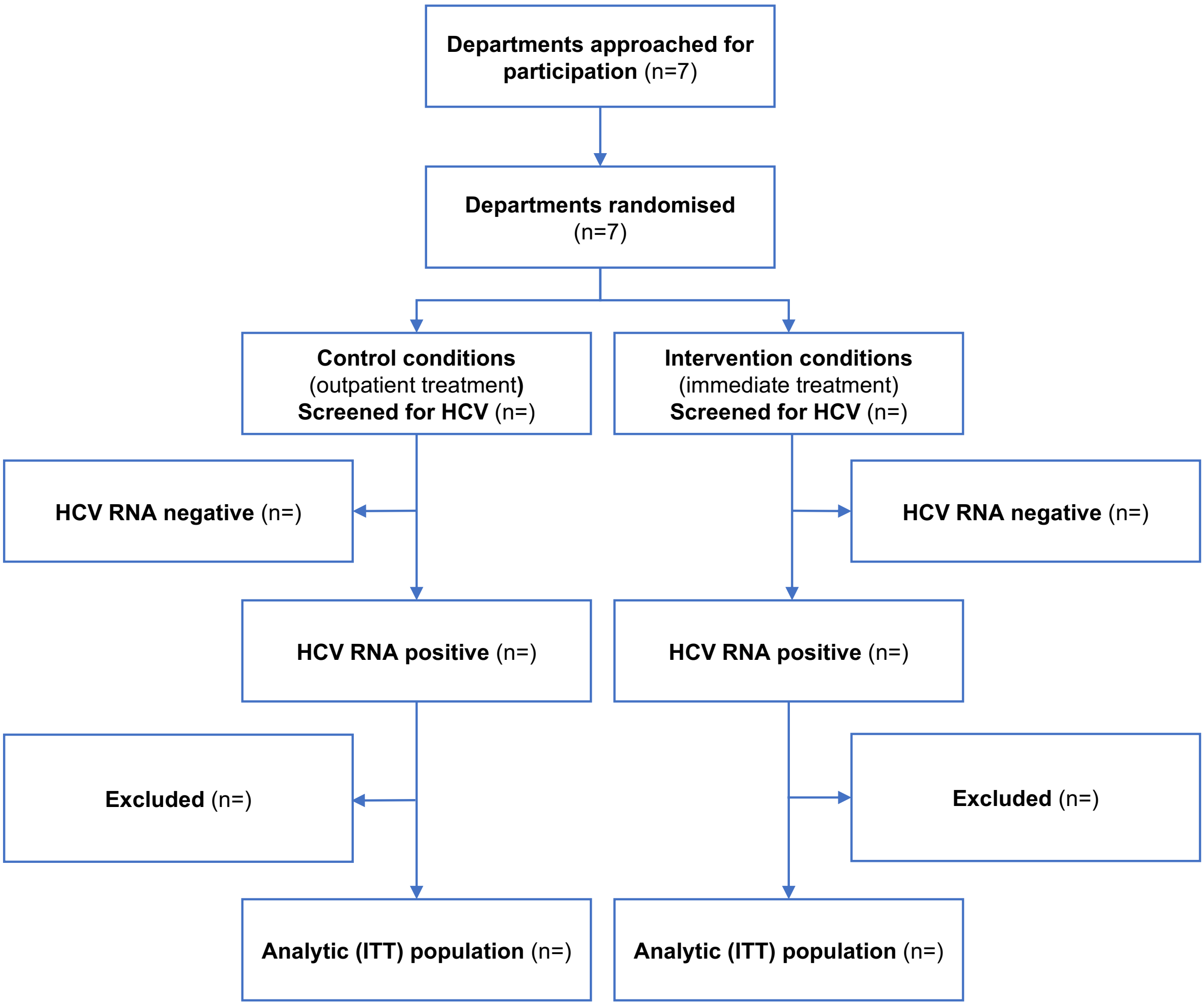

Figure 1. CONSORT diagram showing flow of study participants in OPPORTUNI-C.

**Period**	**1**	**2**	**3**	*	**4**	**5**	**6**	**7**	**8**	**Total**
**Cluster period**	2 months	2 months	2 months		4 months	4 months	3 months	4 months	5 months	**26 months**
**Cluster 1**<br>*Internal medicine AHUS*	Screened:<br>Viremic<br>Enrolled:	Screened:<br>Viremic:<br>Enrolled:	Screened:<br>Viremic:<br>Enrolled:		Screened:<br>Viremic:<br>Enrolled:	Screened:<br>Viremic:<br>Enrolled:	Screened:<br>Viremic:<br>Enrolled:	Screened:<br>Viremic:<br>Enrolled:	Screened:<br>Viremic:<br>Enrolled:	**Screened:<br>Viremic:<br>Enrolled:**
**Cluster 2**<br>*Addiction medicine AHUS*	Screened:<br>Viremic:<br>Enrolled:	Screened:<br>Viremic:<br>Enrolled:	Screened:<br>Viremic:<br>Enrolled:		Screened:<br>Viremic:<br>Enrolled:	Screened:<br>Viremic:<br>Enrolled:	Screened:<br>Viremic:<br>Enrolled:	Screened:<br>Viremic:<br>Enrolled:	Screened:<br>Viremic:<br>Enrolled:	**Screened:<br>Viremic:<br>Enrolled:**
**Cluster 3**<br>*Psychiatry AHUS*	Screened:<br>Viremic:<br>Enrolled:	Screened:<br>Viremic:<br>Enrolled:	Screened:<br>Viremic:<br>Enrolled:		Screened:<br>Viremic:<br>Enrolled:	Screened:<br>Viremic:<br>Enrolled:	Screened:<br>Viremic:<br>Enrolled:	Screened:<br>Viremic:<br>Enrolled:	Screened:<br>Viremic:<br>Enrolled:	**Screened:<br>Viremic:<br>Enrolled:**
**Cluster 4**<br>*Internal medicine OUS*	Screened:<br>Viremic:<br>Enrolled:	Screened:<br>Viremic:<br>Enrolled:	Screened:<br>Viremic:<br>Enrolled:		Screened:<br>Viremic:<br>Enrolled:	Screened:<br>Viremic:<br>Enrolled:	Screened:<br>Viremic:<br>Enrolled:	Screened:<br>Viremic:<br>Enrolled:	Screened:<br>Viremic:<br>Enrolled:	**Screened:<br>Viremic:<br>Enrolled:**
**Cluster 5**<br>*Psychiatry LDS*	Screened:<br>Viremic:<br>Enrolled:	Screened:<br>Viremic:<br>Enrolled:	Screened:<br>Viremic:<br>Enrolled:		Screened:<br>Viremic:<br>Enrolled:	Screened:<br>Viremic:<br>Enrolled:	Screened:<br>Viremic:<br>Enrolled:	Screened:<br>Viremic:<br>Enrolled:	Screened:<br>Viremic:<br>Enrolled:	**Screened:<br>Viremic:<br>Enrolled:**
**Cluster 6**<br>*Addiction medicine OUS*	Screened:<br>Viremic:<br>Enrolled:	Screened:<br>Viremic:<br>Enrolled:	Screened:<br>Viremic:<br>Enrolled:		Screened:<br>Viremic:<br>Enrolled:	Screened:<br>Viremic:<br>Enrolled:	Screened:<br>Viremic:<br>Enrolled:	Screened:<br>Viremic:<br>Enrolled:	Screened:<br>Viremic:<br>Enrolled:	**Screened:<br>Viremic:<br>Enrolled:**
**Cluster 7**<br>*Internal medicine LDS*	Screened:<br>Viremic:<br>Enrolled:	Screened:<br>Viremic:<br>Enrolled:	Screened:<br>Viremic:<br>Enrolled:		Screened:<br>Viremic:<br>Enrolled:	Screened:<br>Viremic:<br>Enrolled:	Screened:<br>Viremic:<br>Enrolled:	Screened:<br>Viremic:<br>Enrolled:	Screened:<br>Viremic:<br>Enrolled:	**Screened:<br>Viremic:<br>Enrolled:**
**Total**	Screened:<br>Viremic:<br>Enrolled:	Screened:<br>Viremic:<br>Enrolled:	Screened:<br>Viremic:<br>Enrolled:		Screened:<br>Viremic:<br>Enrolled:	Screened:<br>Viremic:<br>Enrolled:	Screened:<br>Viremic:<br>Enrolled:	Screened:<br>Viremic:<br>Enrolled:	Screened:<br>Viremic:<br>Enrolled:	**Screened:<br>Viremic:<br>Enrolled:**
**Inclusion rate**										
**HCV RNA prev**										

Table 1. Diagram showing participant recruitment and estimated HCV RNA prevalence according to the stepped-wedge cluster randomised trial design of OPPORTUNI-C. Blank cells represent clusters in control conditions and shaded cells represent clusters in intervention conditions. *One month pause due to COVID-19 lockdown.

Baseline characteristic, n (%)	All (n=)	Intervention (n=)	Control (n=)	Internal medicine (n=)			Addiction medicine (n=)		Psychiatry (n=)	
				#1 AHUS (n=)	#4 OUS (n=)	#7 LDS (n=)	#2 AHUS (n=)	#6 OUS (n=)	#3 AHUS (n=)	#5 LDS (n=)
Age, mean (SD)										
**Age groups**<br>20-29<br>30-39<br>40-49<br>50-59<br>60-80										
**Gender**<br>Male<br>Female										
**Housing status**<br>Rented/owned accommodation<br>Drug rehabilitation institution<br>Low-threshold institution<br>Prison<br>Homeless/on the street										
**Source of income**<br>Part- or full-time job<br>Welfare pension<br>Social<br>Other										
**History of injecting drug use**<br>Yes<br>No										
**Recent (past 3 months) injecting drug use**<br>Yes<br>No										
**Preferred injected drug**<br>Heroin<br>Amphetamines<br>Other										
**Recent (past 3 month) sharing of injecting equipment**										

Yes<br>No										
**Opioid agonist treatment**<br>Yes<br>No										
**Opioid agonist treatment drug**<br>Methadone<br>Buprenorphine<br>Buprenorphine-naloxone<br>Other										
**Stage of liver disease**<br>Mild or no liver fibrosis<br>Significant fibrosis<br>Compensated cirrhosis<br>Decompensated cirrhosis										
FIB-4 index, mean (SD)										
Liver stiffness (kPa), mean (SD)										
**Hepatocellular carcinoma**<br>Yes<br>No/unknown										
**Renal function**<br>eGFR < 30 ml/min/1.73 m^2<br>eGFR 30-59 ml/min/1.73 m^2<br>eGFR >60 ml/min/1.73 m^2										
**HIV co-infection**<br>Yes<br>No<br>Unknown										
**HBV co-infection (HBsAg+)**<br>Yes<br>No<br>Unknown										
**HCV genotype**<br>Not genotyped<br>Genotype 1<br>Genotype 2<br>Genotype 3										

Duration of hospitalization (days), mean (SD)										
**Main discharge diagnosis**<br>Drug-related<br>Alcohol-related<br>Mental health<br>Infectious diseases<br>Hepatology<br>Nephrology<br>Cardiology<br>Other										
**Charlson comorbidity index**<br>1<br>2<br>3<br>≥4										
Charlson comorbidity index, mean (SD)										

Table 2. Characteristics at baseline for study participants in OPPORTUNI-C summarized by randomisation group, cluster and total. Numbers are shown as n (%) unless otherwise indicated.

Outcome	Events, n (%)			Absolute difference, % (95% CI)	Effect size	Cluster effects
	All (n=)	Intervention (n=)	Control (n=)		OR* or HR§ (95% CI)	SD (95% CI)
**Primary outcome** Treatment completion					*	
**Secondary outcome** Treatment initiation					§	

* Estimates derived from mixed effects logistic regression analysis with intervention and calendar time as fixed effects and department as random effect
§ Estimates derived from Cox regression analysis adjusted for calendar time with department as shared frailty variable

Table 3. Summary statistics and estimated effect sizes for the primary and secondary outcome.

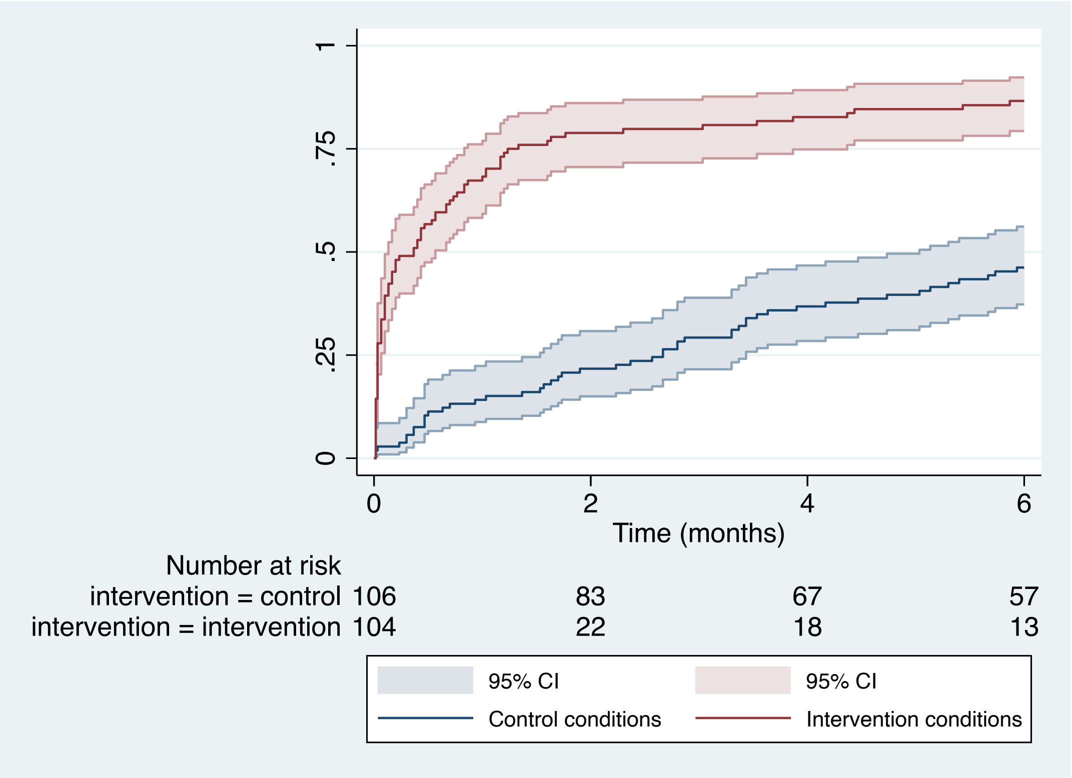

Figure 2. Kaplan-Meier analysis of time to treatment initiation within (A) 6 months and (B) 12 months after enrolment (not shown here). Blue lines represent the proportion of participants in control conditions and red lines represent the proportion of participants in intervention conditions.

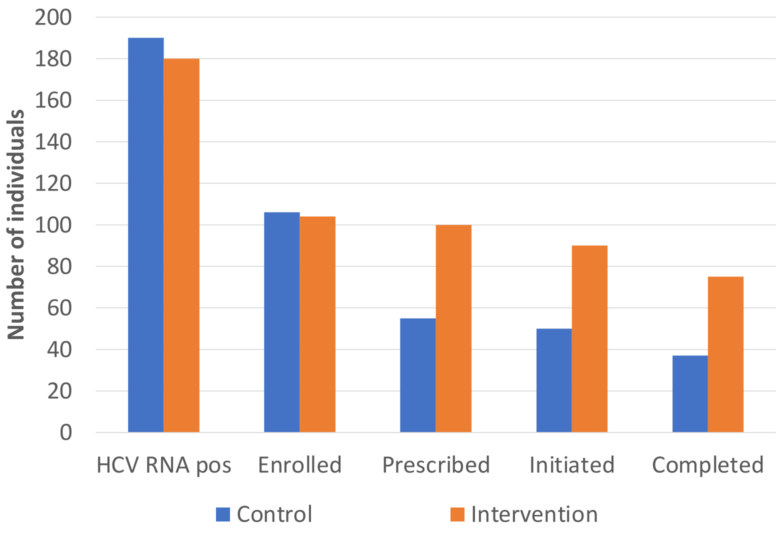

Figure 3. The cascade of care for HCV RNA positive individuals (A) at 6 months following enrolment and (B) at data lock (not shown here), showing the number of participants with enrolment, prescription, initiation, and completion of treatment according to control and intervention conditions.

**Characteristic, n (%)**	**All (n=)**	**Control (n=)**	**Intervention (n=)**
Mean time to treatment (SD)			
**DAA regimen**<br>Sofosbuvir/velpatasvir (12 wks)<br>Sofosbuvir/ledipasvir (12 wks)<br>Grazoprevir/elbasvir (12 wks)<br>Glecaprevir/pibrentasvir (8 wks)			
**Follow-up model**<br>Self-administered<br>Outpatient clinic<br>Low-threshold services<br>Institution/nursing home<br>Municipal nursing services<br>General practitioner			

Table 4. Treatment characteristics for participants who initiated treatment in OPPORTUNI-C.

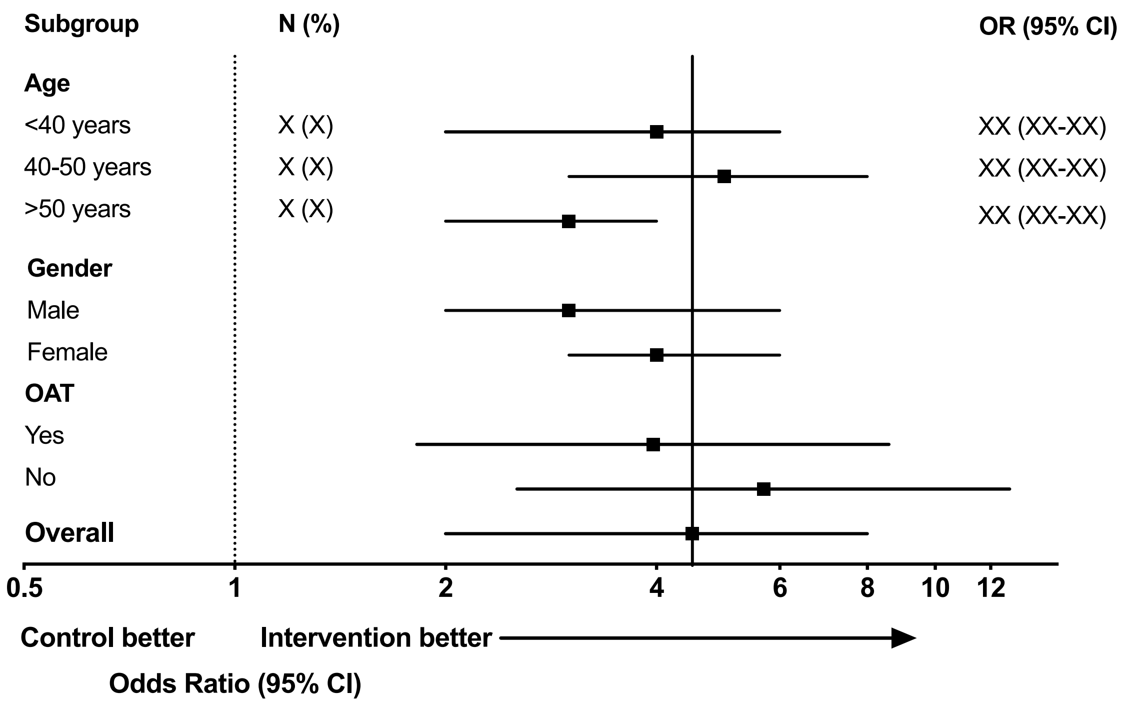

Fig 4. Forest plot of subgroup analyses of the primary outcome. Only selected subgroups are illustrated here.

P intervention	P control	Delta P	N	Cluster size	Power
0.5	0.1	0.4	168	3	0.93
0.5	0.2	0.3	224	4	0.84
0.5	0.2	0.3	168	3	0.74
0.5	0.3	0.2	224	4	0.51
0.6	0.2	0.4	168	3	0.94
0.6	***0.3***	***0.3***	***224***	***4***	***0.86***
0.6	0.4	0.2	224	4	0.52
0.7	0.3	0.4	168	3	0.97
0.7	0.4	0.3	168	3	0.81
0.7	0.5	0.2	224	4	0.58
0.8	0.4	0.4	168	3	0.99
0.8	0.5	0.3	168	3	0.90
0.8	0.6	0.2	224	4	0.70

Supplementary Table 1. Sensitivity analysis showing power and sample size estimates for different assumptions for effect size. Acceptable alternatives with estimated power >0.80 are shown in green, while unsuitable alternatives are shown in red.

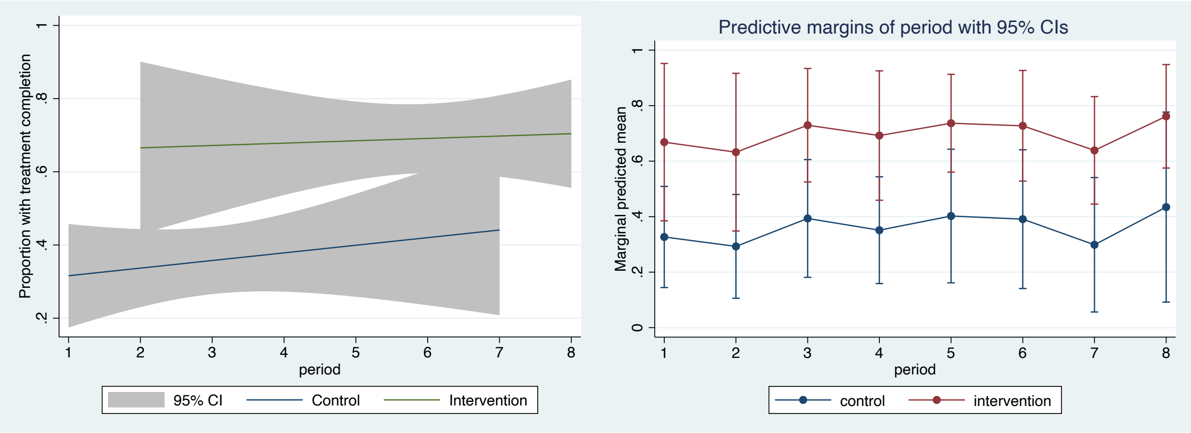

Supplementary Figure 1. Model-based estimate for underlying temporal trends in the primary outcome over duration of the trial for participants in control conditions and intervention conditions.

8. References

1. Midgard H, Finbraten AK, Malme KB, Berg-Pedersen RM, Tanum L, Olsen IC, et al. Opportunistic treatment of hepatitis C virus infection (OPPORTUNI-C): study protocol for a pragmatic stepped wedge cluster randomized trial of immediate versus outpatient treatment initiation among hospitalized people who inject drugs. Trials. 2020;21(1):524.
2. Dore GJ, Ward J, Thursz M. Hepatitis C disease burden and strategies to manage the burden (Guest Editors Mark Thursz, Gregory Dore and John Ward). Journal of viral hepatitis. 2014;21 Suppl 1:1-4.
3. Hajarizadeh B, Grebely J, Dore GJ. Epidemiology and natural history of HCV infection. Nature reviews Gastroenterology & hepatology. 2013;10(9):553-62.
4. WHO. Global progress report on HIV, viral hepatitis and sexually transmitted infections, 2021 - Accountability for the global health sector strategies 2016–2021: Actions for impact. https://www.who.int/publications/i/item/9789240027077. 2021.
5. Degenhardt L, Charlson F, Stanaway J, Larney S, Alexander LT, Hickman M, et al. Estimating the burden of disease attributable to injecting drug use as a risk factor for HIV, hepatitis C, and hepatitis B: findings from the Global Burden of Disease Study 2013. Lancet Infect Dis. 2016;16(12):1385-98.
6. WHO. WHO Global Health Sector Strategy On Viral Hepatitis 2016–2021. http://apps.who.int/iris/bitstream/10665/246177/1/WHO-HIV-2016.06-eng.pdf?ua=1. 2016.
7. Gotte M, Feld JJ. Direct-acting antiviral agents for hepatitis C: structural and mechanistic insights. Nature reviews Gastroenterology & hepatology. 2016;13(6):338-51.
8. Dore GJ, Altice F, Litwin AH, Dalgard O, Gane EJ, Shibolet O, et al. Elbasvir–grazoprevir to treat hepatitis C virus infection in persons receiving opioid agonist therapy: a randomized trial. Annals of internal medicine. 2016;165(9):625-34.
9. Grebely J, Dalgard O, Conway B, Cunningham EB, Bruggmann P, Hajarizadeh B, et al. Sofosbuvir and velpatasvir for hepatitis C virus infection in people with recent injection drug use (SIMPLIFY): an open-label, single-arm, phase 4, multicentre trial. The Lancet Gastroenterology & Hepatology. 2018;3(3):153-61.
10. Grebely J, Conway B, Cunningham EB, Fraser C, Moriggia A, Gane E, et al. Paritaprevir, ritonavir, ombitasvir, and dasabuvir with and without ribavirin in people with HCV genotype 1 and recent injecting drug use or receiving opioid substitution therapy. The International journal on drug policy. 2018;62:94-103.
11. Hajarizadeh B, Cunningham EB, Reid H, Law M, Dore GJ, Grebely J. Direct-acting antiviral treatment for hepatitis C among people who use or inject drugs: a systematic review and meta-analysis. Lancet Gastroenterol Hepatol. 2018;3(11):754-67.
12. Fadnes LT, Aas CF, Vold JH, Leiva RA, Ohldieck C, Chalabianloo F, et al. Integrated treatment of hepatitis C virus infection among people who inject drugs: A multicenter randomized controlled trial (INTRO-HCV). PLoS Med. 2021;18(6):e1003653.
13. Midgard H, Ulstein K, Backe O, Foshaug T, Sorli H, Vennesland K, et al. Hepatitis C treatment and reinfection surveillance among people who inject drugs in a low-threshold program in Oslo, Norway. The International journal on drug policy. 2021:103165.
14. Akiyama MJ, Norton BL, Arnsten JH, Agyemang L, Heo M, Litwin AH. Intensive Models of Hepatitis C Care for People Who Inject Drugs Receiving Opioid Agonist Therapy: A Randomized Controlled Trial. Ann Intern Med. 2019;170(9):594-603.
15. Cunningham EB, Hajarizadeh B, Amin J, Litwin AH, Gane E, Cooper C, et al. Adherence to Once-daily and Twice-daily Direct-acting Antiviral Therapy for Hepatitis C Infection Among People With Recent Injection Drug Use or Current Opioid Agonist Therapy. Clinical infectious diseases : an official publication of the Infectious Diseases Society of America. 2020;71(7):e115-e24.

16. Hajarizadeh B, Cunningham EB, Valerio H, Martinello M, Law M, Janjua NZ, et al. Hepatitis C reinfection following successful antiviral treatment among people who inject drugs: A systematic review and meta-analysis. EASL International Liver Congress 2019, Vienna, Austria. 2019.
17. Grebely J, Bruneau J, Lazarus JV, Dalgard O, Bruggmann P, Treloar C, et al. Research priorities to achieve universal access to hepatitis C prevention, management and direct-acting antiviral treatment among people who inject drugs. The International journal on drug policy. 2017;47:51-60.
18. Grebely J, Hajarizadeh B, Lazarus JV, Bruneau J, Treloar C, International Network on Hepatitis in Substance U. Elimination of hepatitis C virus infection among people who use drugs: Ensuring equitable access to prevention, treatment, and care for all. The International journal on drug policy. 2019;72:1-10.
19. Bruggmann P, Litwin AH. Models of care for the management of hepatitis C virus among people who inject drugs: one size does not fit all. Clinical infectious diseases : an official publication of the Infectious Diseases Society of America. 2013;57 Suppl 2:S56-61.
20. Day E, Hellard M, Treloar C, Bruneau J, Martin NK, Ovrehus A, et al. Hepatitis C elimination among people who inject drugs: Challenges and recommendations for action within a health systems framework. Liver international : official journal of the International Association for the Study of the Liver. 2018.
21. McDonald SA, Hutchinson SJ, Innes HA, Allen S, Bramley P, Bhattacharyya D, et al. Attendance at specialist hepatitis clinics and initiation of antiviral treatment among persons chronically infected with hepatitis C: examining the early impact of Scotland's Hepatitis C Action Plan. Journal of viral hepatitis. 2014;21(5):366-76.
22. Wade AJ, Doyle JS, Gane E, Stedman C, Draper B, Iser D, et al. Outcomes of treatment for hepatitis C in primary care compared to hospital-based care: a randomised controlled trial in people who inject drugs. Clinical infectious diseases : an official publication of the Infectious Diseases Society of America. 2019.
23. Bajis S, Dore GJ, Hajarizadeh B, Cunningham EB, Maher L, Grebely J. Interventions to enhance testing, linkage to care and treatment uptake for hepatitis C virus infection among people who inject drugs: A systematic review. The International journal on drug policy. 2017;47:34-46.
24. Yehia BR, Schranz AJ, Umscheid CA, Lo Re V, 3rd. The treatment cascade for chronic hepatitis C virus infection in the United States: a systematic review and meta-analysis. PloS one. 2014;9(7):e101554.
25. Valerio H, Alavi M, Law M, McManus H, Tillakeratne S, Bajis S, et al. Opportunities to enhance linkage to hepatitis C care among hospitalised people with recent drug dependence in New South Wales, Australia: A population-based linkage study. Clinical infectious diseases : an official publication of the Infectious Diseases Society of America. 2021.
26. Hemming K, Haines TP, Chilton PJ, Girling AJ, Lilford RJ. The stepped wedge cluster randomised trial: rationale, design, analysis, and reporting. Bmj. 2015;350:h391.
27. Hemming K, Taljaard M, Grimshaw J. Introducing the new CONSORT extension for stepped-wedge cluster randomised trials. Trials. 2019;20(1):68.
28. Califf RM, Sugarman J. Exploring the ethical and regulatory issues in pragmatic clinical trials. Clin Trials. 2015;12(5):436-41.
29. The Stata Journal 14. Number 2, pp 363–380 2014.
30. Hemming K, Taljaard M. Sample size calculations for stepped wedge and cluster randomised trials: a unified approach. J Clin Epidemiol. 2016;69:137-46.
31. Hemming K, Taljaard M, Forbes A. Analysis of cluster randomised stepped wedge trials with repeated cross-sectional samples. Trials. 2017;18(1):101.
