# Supplementary material for: Opportunistic Treatment of Hepatitis C Infection Among Hospitalized People Who Inject Drugs (OPPORTUNI-C): A Stepped Wedge Cluster Randomized Trial
Source: Clin Infect Dis. 2023 Nov 22;78(3):582–90. doi: 10.1093/cid/ciad711 (PMC10954343; doi:10.1093/cid/ciad711)
Supplement: ciad711_Supplementary_Data [file ciad711_supplementary_data.zip › supplementary materials_midgard.docx]

**SUPPLEMENTARY MATERIALS**

Opportunistic treatment of hepatitis C infection among hospitalized people who inject drugs (OPPORTUNI-C): A stepped wedge cluster randomized trial

**Authors:**

Håvard Midgard PhD^1,2^, Kristian B. Malme MD^2,13^, Charlotte M. Pihl MD^3,4^, Riikka M. Berg-Pedersen MD^5^, Lars Tanum PhD^6,7^, Ingvild Klundby MSc^8^, Anne Haug MD^9^, Ida Tveter MD^10^, Ronny Bjørnestad^11^, Inge C. Olsen PhD^12^, Ane-Kristine Finbråten PhD^*3,4^, and Olav Dalgard PhD^*2,13^.

^*^Shared last authorship

^1^Department of Gastroenterology, Oslo University Hospital, Norway

^2^Department of Infectious Diseases, Akershus University Hospital, Norway

^3^Department of Medicine, Lovisenberg Diaconal Hospital, Norway

^4^Unger-Vetlesen Institute, Lovisenberg Diaconal Hospital, Norway

^5^Department of Addiction Medicine, Oslo University Hospital, Norway

^6^Department for Research and Development in Mental Health, Akershus University Hospital, Norway

^7^Oslo Metropolitan University, Norway

^8^Department of Microbiology, Oslo University Hospital, Norway

^9^Department of Acute Medicine, Oslo University Hospital, Norway

^10^Department of Infectious Diseases, Oslo University Hospital, Norway

^11^ProLAR Nett, Søgne, Norway

^12^Department of Research Support for Clinical Trials, Oslo University Hospital, Norway

^13^Institute of clinical Medicine, University of Oslo, Norway

**TABLE OF CONTENTS**

Methods (complete methods description) page 3

Supplementary figure legends (1-4) page 12

Supplementary table 1: Power and sample size page 13

Supplementary table 2: Participant recruitment by cluster and period page 14

Supplementary table 3: Baseline characteristics by period page 15

Supplementary table 4: Baseline characteristics by cluster page 18

Supplementary table 5. Main discharge diagnoses page 21

Supplementary table 6: Treatment characteristics page 22

Supplementary table 7: Virologic outcomes page 23

Supplementary table 8: Mortality page 24

References page 25

**METHODS**

*Study design*

OPPORTUNI-C was a pragmatic, open-label, multicenter, stepped wedge cluster randomized trial. The stepped wedge design involved a sequential rollout of the intervention over eight time periods. Seven departments (clusters) of internal medicine (n=3), addiction medicine (n=2), and psychiatry (n=2) at three hospitals in Oslo, Norway, were assigned to change from control (standard of care) to intervention conditions in a random order until all clusters were exposed to the intervention (Supplementary figure 1). The trial commenced on 1 October 2019, and the planned duration of each period was two months. The study protocol has been published previously (1).

We used a pragmatic clinical trial design to mimic ordinary clinical practice as closely as possible and generate optimal conditions for generalizability. This involved broad eligibility criteria, use of clinical infrastructures, extraction of routinely collected data, and analysis according to an intention-to-treat principle.

The study was approved by the Regional Committee for Medical Research Ethics in Norway on 3 March 2019 (reference number 2019-128). The study was conducted according to the Declaration of Helsinki and International Conference on Harmonization Good Clinical Practice guidelines. Written, informed consent was obtained from all participants.

*Clusters*

There were no strict pre-specified cluster eligibility criteria. The selection was based on the clinical experience that individuals with HCV infection in the larger Oslo area often are admitted to these departments, collectively covering a population of approximately one million inhabitants.

The participating clusters were the following:

- *Akershus University Hospital, Lørenskog, Norway:* Department of medicine (Cluster 1)*,* Department of addiction medicine (Cluster 2)*,* Department of psychiatry (Cluster 3)
- *Oslo University Hospital, Oslo, Norway:* Department of medicine (Cluster 4), Department of addiction medicine (Cluster 6)
- *Lovisenberg Diaconal Hospital, Oslo, Norway:* Department of medicine (Cluster 7), Department of psychiatry (Cluster 5)

The three medical departments (cluster 1, 4 and 7) all have referral centers for HCV infection (see control conditions). No referral center provided low-threshold primary HCV care, but the low-threshold HCV clinic in Oslo is accessible for all people who inject drugs (see intervention conditions).

*Participants*

Participant inclusion criteria were 1) age > 18 years, 2) current HCV infection, defined as detectable HCV RNA, 3) admitted for inpatient care in one of the clusters, and 4) able to provide informed written consent. Participants were ineligible only if they 1) had ongoing HCV treatment, 2) were pregnant or breastfeeding, or 3) did not provide or withdrew their consent. Participants with decompensated cirrhosis or previous treatment experience were eligible for inclusion.

*Screening for HCV infection*

An HCV awareness campaign targeting local nurses and physicians was launched in all clusters six months prior to trial commencement. The campaign included oral lectures, electronic newsletters, flyers, and posters presenting key aspects of HCV care and specific information about the trial. According to Norwegian recommendations, all participants with injecting drug use should be tested for HCV infection when admitted for inpatient specialist care. Increasing adherence these guidelines among inpatient staff was a key component of this campaign. Testing activity increased after the implementation of the campaign (2).

Screening for HCV infection (either anti-HCV with reflexive HCV RNA testing or initial HCV RNA testing) was done according to usual practice and as soon as possible after admission. Untargeted screening was performed in clusters of addiction medicine and psychiatry, while risk-based screening targeting individuals with a history of injecting drug use and individuals from high-prevalence countries was performed in clusters of internal medicine. Following identification of any HCV RNA positive individual, the local microbiology department alerted a local investigator who obtained informed consent and facilitated enrolment in cooperation with the clinical hospital staff.

HCV RNA was analyzed twice weekly at the local department of Microbiology using either the COBAS® AmpliPrep/COBAS®TaqMan® HCV Test, v2.0 (Akershus University Hospital) or the COBAS® HCV Test/COBAS® 6800/8800 Systems (Oslo University Hospital). Both laboratories provided reflexive HCV RNA testing. Standard biochemical analyses (hepatitis B and HIV serology, hematology, liver biochemistry, and renal function) were performed as part of usual clinical practice. HCV genotyping was not routinely performed but could have been performed as part of outpatient care.

*Randomization*

Allocation was computer-generated and stratified according to expected cluster size to keep high HCV prevalence clusters separated regarding the timing of the intervention. Clusters of internal medicine (cluster 1, 4 and 7) were assigned ‘large size’, clusters of addiction medicine (cluster 2 and 6) were assigned ‘medium size’ and clusters of psychiatry (cluster 3 and 5) were assigned “small size”. The sequences were prepared by a statistician not involved in enrolment and kept in closed opaque envelopes. Concealment of a new step in the sequence was made available to the researchers on the day of transition and immediately disclosed to the clinical staff at the relevant cluster. The trial was open-label, and all participants, study personnel, and outcome assessors were unblinded to the intervention after concealment.

*Intervention conditions*

During intervention conditions, all participants were offered immediate HCV assessment and treatment initiation during hospitalization or as soon as possible after discharge. The intervention was delivered at both cluster- and individual levels and was maintained throughout the study period after implementation. The intervention was implemented by the local investigator in cooperation with the responsible inpatient physician and relevant clinical staff. This collaboration was considered an important educational aspect of the trial. In Norway, DAA treatment is free of charge for the patients, and the cost is covered by the regional health authorities. Treatment is without restrictions and can be prescribed by any hospital-employed physician using the electronic prescription module in the hospital patient files. The prescribed DAAs can be dispensed from any pharmacy, most often in sequentially delivered four-week packages.

The intervention/model of care comprised of the following components:

1) *Liver disease staging*. Stage of liver disease was assessed based on either liver stiffness measurements (LSM) using transient elastography (FibroScan®) or FIB-4 index (calculated based on age, platelet count, aspartate aminotransferase, and alanine aminotransferase) at the time of enrolment (3). FibroScan® was available on site in all clusters except in the Department of addiction medicine at Oslo University Hospital (Cluster 6), where an ambulant elastography service was available on request. FibroScan® was performed by the primary investigator or a trained nurse. No or mild liver fibrosis was defined as LSM <7 kPa or FIB-4 <1.30. Liver cirrhosis was defined as LSM >12.5 kPa, FIB-4 index >3.25, or presence of typical signs of cirrhosis on imaging. All patients with liver cirrhosis were scored according to the Child-Pugh classification.

2) *Pre-treatment counselling* at the discretion of the treating physician. Standard counseling included disclosure of HCV RNA status, stage of liver disease, prognosis, and potential coinfections, as well as information on potential DAA treatment adverse events and drug–drug interactions. All patients with ongoing injecting drug use received advise on measures to minimize reinfection risk and other harms associated with injecting drug use. All patients received advice on measures to maintain liver health (i.e., alcohol use and metabolic risk factors).

3) *DAA treatment initiation* following Norwegian HCV treatment recommendations (ref). In cases with known HCV genotype, the preferred treatment is genotype-specific with elbasvir/grazoprevir for 8 weeks or sofosbuvir/ledipasvir for 8-12 weeks in genotype 1, or glecaprevir/pibrentasvir for 8 weeks or sofosbuvir/velpatasvir for 12 weeks in genotype 2/3. In cases with unknown genotype, pan-genotypic combinations sofosbuvir/velpatasvir for 12 weeks or glecaprevir/pibrentasvir for 8 weeks is recommended. Following electronic prescription from the treating physician, the DAAs were dispensed from the local pharmacy either directly to the study participants or to an inpatient nurse, depending on the clinical status of the patient.

4) *Individualized follow-up* at the discretion of the treating physician. Many participants are expected to administer the 8–12 weeks treatment without assistance, but some will require closer follow-up with the support from the local low-threshold HCV clinic, relevant institutions or nursing homes, municipal nursing services, or the hospital outpatient clinic. On-treatment HCV RNA monitoring and SVR assessment was scheduled at the discretion of the treating physician in collaboration with the relevant services.

The low-threshold HCV clinic in Oslo is a well-established service from the City of Oslo that has provided ambulant testing, assessment (including FibroScan) and treatment of HCV infection among PWID for the past ten years (3). The clinic is located within the premises of the city’s harm reduction services, which also includes a needle and syringe program, a drug consumption room, short-term emergency accommodation, an outreach team, and a general health clinic. The clinic is staffed by a general practitioner and three nurses with clinical support from an infectious diseases specialist and a hepatologist. The model of care is flexible and individually tailored, employing weekly pillboxes, direct-observed therapy, or self-administration.

Post-treatment hepatocellular carcinoma surveillance was offered for those with evidence of liver cirrhosis according to usual care. Local practice decided if the referral was to the gastroenterology outpatient clinic or to the department of radiology.

*Control conditions*

During control conditions, all enrolled participants were referred for outpatient HCV care following discharge in accordance with the established standard of care for hospitalized individuals. Minimal pre-treatment assessments were done during hospitalization in these individuals. Local practice decided if the referral was to the outpatient clinic for infectious diseases or gastroenterology. At Akershus University Hospital, referral was to the department of infectious diseases regardless of stage of liver disease. At Oslo University Hospital and Lovisenberg Diaconal Hospital, referral was to the departments of infectious diseases for non-cirrhotic individuals and to the departments of gastroenterology for cirrhotic individuals. All referral centers offered individualized HCV care in line with the Norwegian HCV guidelines including adherence and laboratory monitoring during treatment. No referral centers offered patient navigation or directly observed therapy.

*Baseline data*

Participants did not complete a conventional case report form. Instead, the following socio-demographic background variables and clinical data were summarized in a standardized inclusion template in the electronic patient files at enrolment:

- Employment status (part- or full-time job, welfare pension, social benefits, other)
- Housing status (rented/owned accommodation, drug rehabilitation institution, low-threshold institution, prison, homeless/on the street)
- Ever and recent (past 3 months) injecting drug use
- Preferred injected drug (heroin, amphetamines, other, mixed)
- Recent injecting risk behaviors (sharing of needles, syringes, or ancillary equipment)
- Current opioid agonist therapy (methadone, buprenorphine, buprenorphine-naloxone, other)
- Stage of liver disease (liver stiffness measurements, FIB4-index)
- Renal function (estimated glomerular filtration rate)
- Any relevant coinfections (HIV, HBV)

*Outcomes*

The primary outcome was *treatment completion*, defined as dispensing the final four-week package of the prescribed DAAs from the pharmacy within six months after enrolment. Failure to accomplish the primary outcome was noted either if no treatment had been dispensed (i.e., loss to follow-up or other reasons), if treatment had been dispensed but completed later than six months after enrolment (i.e., delayed treatment initiation), or if the final package had not been dispensed (i.e., treatment discontinuation).

The first secondary outcome was *treatment initiation*, defined as dispensing the first package of DAAs within six months after enrolment. The time at risk for each participant was from the date of enrolment until the date of treatment, death, or six months after enrolment, whatever came first.

The final secondary outcome was *sustained virologic response (SVR),* defined as undetectable HCV RNA at least four weeks after the estimated date of end of treatment (SVR$\geq$4). Failure to achieve SVR was noted either if HCV RNA was detectable following end of treatment (i.e., virologic failure), if no samples were available for SVR assessment (i.e., loss to follow-up), or if no DAAs were dispensed (i.e., no treatment).

*Data extraction*

Data on treatment completion and treatment initiation were extracted retrospectively by review of the ‘core medical record’ in the electronic patient files six months after enrolment of the final participant. This record contains complete prescription and dispensation data from pharmacies nationwide within the previous three years for all individuals with a Norwegian social security number. Although most patients receive DAAs in sequentially dispensed four-week packages, the date of treatment completion was estimated for those who were dispensed the whole treatment course at once.

Data on SVR, baseline variables, and causes of death were obtained by retrospective review of the electronic hospital files, including the standardized inclusion template and microbiology files from local and collaborating laboratories. Charlson Comorbidity Index (4) was calculated retrospectively. No measures of adherence or records of protocol deviations were recorded. The number of HCV screening tests performed during the trial, and the number of HCV RNA positive tests were extracted retrospectively from the microbiology laboratory systems and reported for each cluster-period of the trial. Attrition was calculated based on these data.

*Statistical methods*

To show a 30% difference in effect size (60% intervention vs. 30% control) for the primary outcome, with 85% power and 5% significance level, assuming a large intra-cluster correlation coefficient of 0.2, we planned to recruit on average 4 participants per cluster per period (cluster-period) for a total of 224 participants. Assumptions of effect sizes and intra-cluster correlation were conservative and based on data from clinical databases at Akershus University Hospital and a low-threshold HCV clinic in downtown Oslo (3). A sensitivity analysis including a wider range of estimates for effect sizes is shown in Supplementary table 1. There were no interim analyses or stopping guidelines, and no data monitoring committee.

All trial data were summarized by intervention condition, cluster, and period, and reported as mean (SD), median (interquartile range) or N (%) as appropriate. The outcomes were analyzed following an intention-to-treat principle according to cluster allocation regardless of what occurred, with no account of protocol non-adherence. The analysis population was defined as all participants fulfilling the eligibility criteria. Data lock was set to 30 June 2022 (six months after enrolment of the final participant).

Outcomes are reported as proportions, risk differences, and risk ratios with 95% exact confidence intervals (CI). The primary outcome (treatment completion) was analyzed as a binary variable using mixed-effects logistic regression adjusted for intervention condition and calendar time (months from enrolment) with cluster as random effect, according to the Hussey and Hughes model (5). As robustness analysis, we did a permutation test for the primary outcome with 10 000 random permutations of the cluster allocation (6).

The secondary outcome (treatment initiation) was analyzed as time-to-event using Kaplan Maier analysis and Cox regression adjusted for calendar time with cluster as a shared frailty factor (7). The time at risk for each participant was from the date of enrolment until the date of treatment initiation, death, or six months after enrolment, whatever came first. The proportional hazards assumption was tested using log–log transformation of the failure function and Schoenfeld residuals. As robustness analysis, the Cox model was analyzed without a shared frailty factor, but with the variance estimated using a clustered sandwich estimator (8).

Effect estimates from regression models are presented as adjusted odds ratios (aOR) or adjusted hazard ratios (aHR) with 95% CI, and superiority of the intervention is claimed if a two-sided p-value under the null hypothesis is less than 0.05 in favor of the intervention. Random (cluster) effects are reported as the estimated intra-cluster correlation coefficient and the estimated standard deviation of the intercept on the logit scale.

Because complete prescription data are evident from the ‘core medical record’ there were no missing data on treatment completion and treatment initiation unless participants withdrew their consent. Missing SVR data were handled using worst case imputation and assigned as failure.

Subgroup analyses of the primary and secondary outcomes were performed according to the following pre-specified background variables *a-priory* hypothesized to be associated with the outcomes: age, sex, housing status, recent injecting drug use, opioid agonist therapy, preferred injected drug, liver cirrhosis, Charlson Comorbidity Index, and discipline. Point estimates and 95% CI were calculated using the same regression models with intervention x subgroup interaction.

A post hoc analysis was performed to evaluate the effect of the intervention on mortality. Crude mortality rates with 95% Poisson CI were calculated for intervention and control conditions, and according to treatment initiation and treatment completion. The time at risk for each participant was from the date of enrolment until the date of death or the date of data lock, whatever came first. Overall survival was analyzed using Cox regression adjusted for intervention and calendar time.

All analyses were performed using STATA 17 (College Station, TX, USA) with the sample size calculation using the *steppedwedge* package (9).

**SUPPLEMENTARY FIGURE LEGENDS**

**Supplementary figure 1.** The stepped wedge design schematic for OPPORTUNI-C showing a sequential rollout of the intervention over eight time periods (T1-T8) until all clusters were exposed to the intervention. One cluster (department) was randomly assigned each sequence. Blank cells represent clusters in control conditions and blue cells represent clusters in intervention conditions. The planned duration of each time period was two months. Retrospective data extraction was scheduled six months following enrolment of the final participant.

**Supplementary** **figure 2.** Overview of participant recruitment showing rates of screening, viraemia, and enrolment over the duration of the trial.

**Supplementary** **figure 3.** Viremic rates (i.e., the proportion HCV RNA positive screening tests) in clusters of internal medicine, addiction medicine, and psychiatry over the duration of the trial.

**Supplementary figure 4.** The cascade of care at (A) six months following enrolment and (B) at data lock, showing the number of participants with enrolment, prescription, initiation, and completion of treatment according to intervention and control conditions.

**SUPPLEMENTARY** **TABLES**

| P intervention | P control | Delta P | Sample size | Cluster-period size | Power |
| --- | --- | --- | --- | --- | --- |
| 0.5 | **0.1** | **0.4** | **168** | **3** | **0.93** |
| 0.5 | **0.2** | **0.3** | **224** | **4** | **0.84** |
| 0.5 | 0.2 | 0.3 | 168 | 3 | 0.74 |
| 0.5 | 0.3 | 0.2 | 224 | 4 | 0.51 |
| 0.6 | **0.2** | **0.4** | **168** | **3** | **0.94** |
| *0.6* | ***0.3*** | ***0.3*** | ***224*** | ***4*** | ***0.86*** |
| 0.6 | 0.4 | 0.2 | 224 | 4 | 0.52 |
| 0.7 | **0.3** | **0.4** | **168** | **3** | **0.97** |
| 0.7 | **0.4** | **0.3** | **168** | **3** | **0.81** |
| 0.7 | 0.5 | 0.2 | 224 | 4 | 0.58 |
| 0.8 | **0.4** | **0.4** | **168** | **3** | **0.99** |
| 0.8 | **0.5** | **0.3** | **168** | **3** | **0.90** |
| 0.8 | 0.6 | 0.2 | 224 | 4 | 0.70 |

**Supplementary** **table 1.** Power and sample size estimates for different assumptions for effect size. Acceptable alternatives with estimated power >0.80 are shown in bold.

| **Time period** | **T1** | **T2** | **T3** | ***** | **T4** | **T5** | **T6** | **T7** | **T8** | **Total** |
| --- | --- | --- | --- | --- | --- | --- | --- | --- | --- | --- |
| **Date of transition** | 01 Oct 2019 | 02 Dec 2019 | 03 Feb 2020 |  | 04 May 2020 | 01 Sep 2020 | 04 Jan 2021 | 05 April 2021 | 02 Aug 2021 |  |
| **Duration of period** | 2 months | 2 months | 2 months |  | 4 months | 4 months | 3 months | 4 months | 5 months | **26 months** |
| **Cluster 1:** Internal medicine (Akershus University Hospital) | Screened: 103  Viremic: 8  Enrolled: 3 | Screened: 106  Viremic: 6  Enrolled: 3 | Screened: 77  Viremic: 7  Enrolled: 6 |  | Screened: 151  Viremic: 13  Enrolled: 5 | Screened: 159  Viremic: 11  Enrolled: 4 | Screened: 99  Viremic: 9  Enrolled: 5 | Screened: 178  Viremic: 20  Enrolled: 5 | Screened: 156  Viremic: 8  Enrolled: 6 | **Screened:1029**  **Viremic: 82**  **Enrolled: 37** |
| **Cluster 2:** Addiction medicine (Akershus University Hospital) | Screened: 69  Viremic: 8  Enrolled: 3 | Screened: 70  Viremic: 11  Enrolled: 6 | Screened: 57  Viremic: 2  Enrolled: 2 |  | Screened: 111  Viremic: 6  Enrolled: 2 | Screened: 140  Viremic: 11  Enrolled: 1 | Screened: 129  Viremic: 4  Enrolled: 1 | Screened: 132  Viremic: 4  Enrolled: 2 | Screened: 199  Viremic: 6  Enrolled: 5 | **Screened: 907**  **Viremic: 52**  **Enrolled: 22** |
| **Cluster 3:** Psychiatry (Akershus University Hospital) | Screened: 280  Viremic: 7  Enrolled: 2 | Screened: 278  Viremic: 3  Enrolled: 1 | Screened: 199  Viremic: 2  Enrolled: 1 |  | Screened: 394  Viremic: 7  Enrolled: 3 | Screened: 416  Viremic: 8  Enrolled: 5 | Screened: 341  Viremic: 3  Enrolled: 0 | Screened: 465  Viremic: 5  Enrolled: 1 | Screened: 533  Viremic: 3  Enrolled: 3 | **Screened:2906**  **Viremic: 38**  **Enrolled: 16** |
| **Cluster 4:** Internal medicine (Oslo University Hospital) | Screened: 156  Viremic: 9  Enrolled: 7 | Screened: 146  Viremic: 5  Enrolled: 5 | Screened: 123  Viremic: 5  Enrolled: 2 |  | Screened:247  Viremic: 11  Enrolled: 4 | Screened: 284  Viremic: 10  Enrolled: 9 | Screened: 205  Viremic: 4  Enrolled: 4 | Screened: 244  Viremic: 6  Enrolled: 6 | Screened: 326  Viremic: 2  Enrolled: 1 | **Screened:1731**  **Viremic: 52**  **Enrolled: 38** |
| **Cluster 5:** Psychiatry (Lovisenberg Diaconal Hospital) | Screened: 22  Viremic: 0  Enrolled: 0 | Screened: 37  Viremic: 0  Enrolled: 0 | Screened: 22  Viremic: 1  Enrolled: 1 |  | Screened: 45  Viremic: 1  Enrolled: 1 | Screened: 77  Viremic: 3  Enrolled: 3 | Screened: 206  Viremic: 4  Enrolled: 3 | Screened: 268  Viremic: 1  Enrolled: 1 | Screened: 327  Viremic: 7  Enrolled: 3 | **Screened:1004**  **Viremic: 17**  **Enrolled: 12** |
| **Cluster 6:** Addiction medicine (Oslo University Hospital) | Screened: 49  Viremic: 10  Enrolled: 6 | Screened: 62  Viremic: 8  Enrolled: 5 | Screened: 62  Viremic: 14  Enrolled: 10 |  | Screened: 124  Viremic:12  Enrolled: 2 | Screened: 166  Viremic: 14  Enrolled: 2 | Screened: 93  Viremic: 16  Enrolled: 6 | Screened: 114  Viremic: 15  Enrolled: 8 | Screened: 155  Viremic: 8  Enrolled: 4 | **Screened: 825**  **Viremic: 97**  **Enrolled: 43** |
| **Cluster 7:** Internal medicine (Lovisenberg Diaconal Hospital) | Screened: 91  Viremic: 9  Enrolled: 5 | Screened: 79  Viremic: 7  Enrolled: 4 | Screened: 63  Viremic: 3  Enrolled: 2 |  | Screened: 148  Viremic: 14  Enrolled: 9 | Screened: 123  Viremic: 7  Enrolled: 4 | Screened: 81  Viremic: 4  Enrolled: 2 | Screened: 109  Viremic: 7  Enrolled: 3 | Screened: 145  Viremic: 7  Enrolled: 3 | **Screened: 839**  **Viremic: 58**  **Enrolled: 32** |
| **Total** | Screened: 770  Viremic: 51  Enrolled: 26 | Screened: 778  Viremic: 40  Enrolled: 24 | Screened: 603  Viremic: 34  Enrolled: 24 |  | Screened:1220  Viremic: 64  Enrolled: 26 | Screened:1365  Viremic: 64  Enrolled: 28 | Screened:1154  Viremic: 44  Enrolled: 21 | Screened:1510  Viremic: 58  Enrolled: 26 | Screened: 1841  Viremic: 41  Enrolled: 25 | **Screened:9241**  **Viremic: 396**  **Enrolled: 200** |

**Supplementary** **table 2.** Diagram showing participant recruitment by cluster and period according to the stepped wedge cluster randomized trial design of OPPORTUNI-C. Blank cells represent clusters in control conditions and shaded cells represent clusters in intervention conditions. The numbers of screened and viremic individuals throughout the trial may include some of the same individuals, but the numbers of enrolled participants are unique individuals. *The trial was paused for one month during April 2020 due to Covid-19 lockdown in Norway.

| **Variable** | **Total (n=200)** | **T1 (n=26)** | **T2 (n=24)** | **T3 (n=24)** | **T4 (n=26)** | **T5 (n=28)** | **T6 (n=21)** | **T7 (n=26)** | **T8 (n=25)** |
| --- | --- | --- | --- | --- | --- | --- | --- | --- | --- |
| **Age, mean (SD)** | 47.4 (12.7) | 46.7 (11.5) | 48.2 (11.9) | 45.0 (12.7) | 50.2 (13.8) | 42.7 (11.7) | 48.4 (11.6) | 47.7 (14.6) | 50.8 (13.1) |
| **Age groups**  20-29  30-39  40-49  50-59  60-80 | 21 (10.5)  40 (20.0)  51 (25.5)  52 (26.0)  36 (18.0) | 2 (7.7)  4 (15.4)  9 (34.6)  8 (30.8)  3 (11.5) | 2 (8.3)  7 (29.3)  5 (20.8)  5 (20.8)  5 (20.8) | 3 (12.5)  7 (29.2)  5 (20.8)  6 (25.0)  3 (12.5) | 2 (7.7)  4 (15.4)  9 (34.6)  3 (11.5)  8 (30.8) | 5 (17.9)  7 (25.0)  7 (25.0)  7 (25.0)  2 (7.1) | 1 (4.8)  4 (19.0)  7 (33.3)  6 (28.6)  3 (14.3) | 4 (15.4)  4 (15.4)  3 (11.5)  8 (30.8)  7 (26.9) | 2 (8.0)  3 (12.0)  6 (24.0)  9 (36.0)  5 (20.0) |
| **Sex**  Male  Female | 145 (72.5)  55 (27.5) | 20 (76.9)  6 (23.1) | 19 (79.2)  5 (20.8) | 20 (83.3)  4 (16.7) | 18 (69.2)  8 (30.8) | 20 (71.4)  8 (28.6) | 13 (61.9)  8 (38.1) | 17 (65.4)  9 (34.6) | 18 (72.0)  7 (28.0) |
| **Housing status**  Rented/owned accommodation  Drug rehabilitation institution  Low-threshold institution  Prison  Homeless/on the street | 124 (62.0)  10 (5.0)  28 (14.0)  1 (0.5)  37 (18.5) | 14 (53.8)  2 (7.7)  4 (15.4)  1 (3.8)  5 (19.3) | 16 (66.6)  0 (0.0)  1 (4.2)  0 (0.0)  7 (29.2) | 15 (62.5)  0 (0.0)  4 (16.7)  0 (0.0)  5 (20.8) | 15 (57.7)  1 (3.9)  3 (11.5)  0 (0.0)  7(26.9) | 11 (39.3)  5 (17.9)  8 (28.5)  0 (0.0)  4 (14.3) | 14 (66.7)  1 (4.8)  2 (9.5)  0 (0.0)  4 (19.0) | 21 (80.7)  1 (3.9)  3 (11.5)  0 (0.0)  1 (3.9) | 18 (72.0)  0 (0.0)  3 (12.0)  0 (0.0)  4 (16.0) |
| **Source of income***  Part- or full-time job  Welfare pension  Social  Other | 26 (13.1)  116 (58.6)  52 (26.3)  4 (2.0) | 0 (0.0)  18 (69.2)  8 (30.8)  0 (0.0) | 2 (8.7)  12 (52.2)  9 (39.1)  0 (0.0) | 7 (29.2)  11 (45.8)  6 (25.0)  0 (0.0) | 1 (3.9)  16 (61.5)  8 (30.8)  1 (3.8) | 3 (10.7)  18 (64.3)  6 (21.4)  1 (3.6) | 3 (14.3)  13 (61.9)  4 (19.0)  1 (4.8) | 7 (26.9)  13 (50.0)  6 (23.1)  0 (0.0) | 3 (12.5)  15 (62.5)  5 (20.8)  1 (4.2) |
| **History of injecting drug use**  Yes  No | 183 (91.5)  17 (8.5) | 26 (100.0)  0 (0.0) | 23 (95.8)  1 (4.2) | 22 (91.7)  2 (8.3) | 24 (92.3)  2 (7.7) | 26 (92.9)  2 (7.1) | 18 (85.7)  3 (14.3) | 20 (76.9)  6 (23.1) | 24 (96.0)  1 (4.0) |
| **Recent (past 3 months) injecting drug use**  Yes  No | 121 (60.5)  79 (39.5) | 20 (76.9)  6 (23.1) | 14 (58.3)  10 (41.7) | 13 (54.2)  11 (45.8) | 17 (65.4)  9 (34.6) | 18 (64.3)  10 (35.7) | 9 (42.9)  12 (57.1) | 13 (50.0)  13 (50.0) | 17 (68.0)  8 (32.0) |
| **Recent sharing of injecting equipment**†  Yes  No  Unknown | 34 (28.1)  54 (44.6)  33 (27.3) | 6 (30.0)  8 (40.0)  6 (30.0) | 5 (35.7)  4 (28.6)  5 (35.7) | 5 (38.5)  5 (38.5)  3 (23.0) | 5 (29.4)  10 (58.8)  2 (11.8) | 4 (22.2)  7 (38.9)  7 (38.9) | 2 (22.2)  2 (22.2)  5 (55.6) | 2 (15.4)  8 (61.5)  3 (23.1) | 5 (29.4)  10 (58.8)  2 (11.8) |
| **Preferred injected drug**‡  Heroin  Amphetamines  Other/mixed | 114 (64.8)  51 (29.0)  11 (6.3) | 13 (50.0)  12 (46.1)  1 (3.9) | 16 (76.2)  5 (23.8)  0 (0.0) | 13 (59.1)  4 (18.2)  5 (22.7) | 19 (86.4)  3 (13.6)  0 (0.0) | 19 (79.2)  5 (20.8)  0 (0.0) | 10 (55.6)  7 (38.8)  1 (5.6) | 11 (57.9)  7 (36.8)  1 (5.3) | 13 (54.2)  8 (33.3)  3 (12.5) |
| **Current opioid agonist therapy**  Yes  No | 90 (45.0)  110 (55.0) | 14 (53.8)  12 (46.2) | 10 (41.7)  14 (58.3) | 9 (37.5)  15 (62.5) | 17 (65.4)  9 (34.6) | 15 (53.6)  13 (46.4) | 7 (33.3)  14 (66.7) | 10 (38.5)  16 (61.5) | 8 (32.0)  17 (68.0) |
| **Opioid agonist therapy drug**§  Methadone  Buprenorphine  Buprenorphine-naloxone  Other | 48 (53.3)  36 (40.0)  2 (2.2)  4 (4.4) | 9 (64.3)  4 (28.6)  1 (7.1)  0 (0.0) | 5 (50.0)  4 (40.0)  0 (0.0)  1 (10.0) | 4 (44.5)  5 (55.5)  0 (0.0)  0 (0.0) | 7 (41.2)  9 (52.9)  0 (0.0)  1 (5.9) | 6 (40.0)  7 (46.7)  0 (0.0)  2 (13.3) | 3 (42.9)  3 (42.9)  1 (14.2)  0 (0.0) | 8 (80.0)  2 (20.0)  0 (0.0)  0 (0.0) | 6 (75.0)  2 (25.0)  0 (0.0)  0 (0.0) |
| **Stage of liver disease**¶  Mild or no liver fibrosis  Intermediate fibrosis  Compensated cirrhosis  Decompensated cirrhosis | 102 (52.0)  54 (27.6)  21 (10.7)  19 (9.7) | 13 (56.5)  6 (26.1)  3 (13.0)  1 (4.4) | 12 (50.0)  9 (37.5)  1 (4.2)  2 (8.3) | 13 (54.2)  4 (16.7)  3 (12.5)  4 (16.6) | 11 (44.0)  8 (32.0)  1 (4.0)  5 (20.0) | 14 (50.0)  10 (35.7)  1 (3.6)  3 (10.7) | 11 (52.4)  6 (28.6)  3 (14.3)  1 (4.7) | 13 (50.0)  4 (15.4)  6 (23.1)  3 (11.5) | 15 (60.0)  7 (28.0)  3 (12.0)  0 (0.0) |
| **FIB-4 index, mean (SD)**** | 2.72 (5.95) | 2.93 (4.16) | 2.57 (3.57) | 5.36 (15.0) | 3.02 (3.36) | 2.09 (2.48) | 1.89 (1.46) | 2.46 (2.97) | 1.46 (1.85) |
| **Hepatocellular carcinoma**  Yes  No or not assessed | 9 (4.5)  191 (95.5) | 1 (3.8)  25 (96.2) | 1 (4.2)  23 (95.8) | 2 (8.3)  22 (91.7) | 0 (0.0)  26 (100.0) | 0 (0.0)  28 (100.0) | 2 (9.5)  19 (90.5) | 3 (11.5)  23 (88.5) | 0 (0.0)  25 (100.0) |
| **Renal function**  eGFR >60 ml/min/1.73 m^2^  eGFR 30-59 ml/min/1.73 m^2^  eGFR < 30 ml/min/1.73 m^2^ | 188 (94.0)  8 (4.0)  4 (2.0) | 26 (100.0)  0 (0.0)  0 (0.0) | 24 (100.0)  0 (0.0)  0 (0.0) | 24 (100.0)  0 (0.0)  0 (0.0) | 22 (84.6)  3 (11.5)  1 (3.9) | 26 (92.9)  0 (0.0)  2 (7.1) | 17 (80.9)  3 (14.3)  1 (4.8) | 26 (100.0)  0 (0.0)  0 (0.0) | 23 (92.0)  2 (8.0)  0 (0.0) |
| **HIV co-infection**  Yes  No  Not assessed | 6 (3.0)  169 (84.5)  25 (12.5) | 1 (3.85)  21 (8.8)  4 (15.5) | 0 (0.0)  20 (83.3)  4 (16.7) | 0 (0.0)  24 (100.0)  0 (0.0) | 1 (3.9)  22 (84.6)  3 (11.5) | 2 (7.1)  22 (78.6)  4 (14.3) | 1 (4.8)  18 (85.7)  2 (9.5) | 1 (3.9)  22 (84.6)  3 (11.5) | 0 (0.0)  20 (80.0)  5 (20.0) |
| **HBV co-infection (HBsAg+)**  Yes  No  Not assessed | 1 (0.5)  180 (90.0)  19 (9.5) | 0 (0.0)  22 (84.6)  4 (15.4) | 0 (0.0)  21 (87.5)  3 (12.5) | 0 (0.0)  24 (100.0)  0 (0.0) | 0 (0.0)  23 (88.5)  3 (11.5) | 1 (3.6)  23 (82.1)  4 (14.3) | 0 (0.0)  19 (90.5)  2 (9.5) | 0 (0.0)  24 (92.3)  2 (7.7) | 0 (0.0)  24 (96.0)  1 (4.0) |
| **HCV genotype**  Genotype 1  Genotype 2  Genotype 3  Genotype 4-6  Not genotyped | 47 (23.5)  7 (3.5)  48 (24.0)  6 (3.0)  92 (46.0) | 8 (30.8)  1 (3.9)  7 (26.9)  0 (0.0)  10 (38.4) | 8 (33.3)  2 (8.3)  6 (25.0)  1 (4.2)  7 (29.2) | 6 (25.0)  1 (4.2)  11 (45.8)  0 (0.0)  6 (25.0) | 9 (34.6)  1 (3.9)  5 (19.2)  0 (0.0)  11 (42.3) | 7 (25.0)  0 (0.0)  3 (10.7)  2 (7.2)  16 (57.1) | 4 (19.1)  0 (0.0)  6 (28.6)  2 (9.5  9 (42.8) | 4 (15.4)  1 (3.9)  6 (23.1)  1 (3.9)  14 (53.8) | 1 (4.0)  1 (4.0)  4 (16.0)  0 (0.0)  19 (76.0) |
| **Days of hospitalization, median (IQR)** | 6 (3-13) | 5 (3-7) | 6 (3-12) | 8.5 (4-12.5) | 5.5 (1-13) | 9.5 (2.5-19.5) | 8 (4-15) | 4 (2-8) | 7 (3-13) |
| **Main discharge diagnosis**  Drug related  Infectious diseases  Gastroenterology/hepatology  Mental health  Cardiopulmonary  Alcohol related  Other | 93 (46.5)  33 (16.5)  24 (12.0)  15 (7.5)  14 (7.0)  7 (3.5)  14 (7.0) | 16 (61.5)  3 (11.5)  3 (11.5)  1 (3.9)  1 (3.8)  0 (0.0)  2 (7.7) | 12 (50.0)  1 (4.2)  3 (12.5)  1 (4.2)  5 (20.8)  1 (4.2)  1 (4.1) | 14 (58.3)  2 (8.3)  5 (20.8)  0 (0.0)  1 (4.2)  1 (4.2)  1 (4.2) | 10 (38.5)  5 (19.2)  4 (15.4)  2 (7.7)  1 (3.8)  0 (0.0)  4 (15.4) | 7 (25.0)  10 (35.7)  2 (7.1)  5 (17.9)  1 (3.6)  2 (7.1)  1 (3.6) | 7 (33.3)  6 (28.6)  2 (9.5)  2 (9.5)  1 (4.7)  2 (9.5)  1 (4.9) | 12 (46.2)  5 (19.2)  2 (7.7)  1 (3.8)  2 (7.7)  1 (3.8)  3 (11.6) | 15 (60.0)  1 (4.0)  3 (12.0)  3 (12.0)  2 (8.0)  0 (0.0)  1 (4.0) |
| **Charlson comorbidity index**  0-1  2-3  4-5  $\geq$6 | 101 (50.5)  43 (21.5)  33 (16.5)  23 (11.5) | 14 (53.9)  5 (19.2)  6 (23.1)  1 (3.8) | 13 (54.2)  4 (16.7)  3 (12.5)  4 (16.6) | 15 (62.5)  2 (8.3)  3 (12.5)  4 (16.7) | 12 (46.2)  6 (23.0)  4 (15.4)  4 (15.4) | 15 (53.6)  10 (35.7)  0 (0.0)  3 (10.7) | 10 (47.6)  5 (23.8)  3 (14.3)  3 (14.3) | 12 (46.1)  1 (3.9)  9 (34.6)  4 (15.4) | 10 (40.0)  10 (40.0)  5 (20.0)  0 (0.0) |
| **Charlson comorbidity index, mean (SD)** | 2.6 (2.3) | 2.3 (1.7) | 2.8 (2.6) | 2.6 (2.5) | 2.8 (2.1) | 2.2 (1.9) | 2.7 (2.1) | 3.4 (3.1) | 2.2 (1.4) |

**Supplementary** **table 3.** Baseline characteristics summarized by time period and total. Numbers are shown as n (%) unless otherwise indicated. Missing values are excluded from percentages.

SD = standard deviation; IQR = interquartile range; FIB-4 = fibrosis-4; GFR = glomerular filtration rate; HIV = human immunodeficiency virus; HBV = hepatitis B virus

* Missing data for 2 participants (1 intervention, 1 control)

† Among those with recent (past 3 months) injecting drug use

‡ Among those with a history of injecting drug use; missing data for 7 participants (2 intervention, 5 control)

§ Among those with current opioid agonist therapy

¶ Based on liver stiffness measurements in 86, FIB-4 index in 107, and imaging in 3 participants; missing data for 4 control participants

** Among 193 participants with an available FIB-4 index

| **Variable** | **Total**  **(n=200)** | **Internal medicine (n=107)** | | | **Addiction medicine (n=65)** | | **Psychiatry (n=28)** | |
| --- | --- | --- | --- | --- | --- | --- | --- | --- |
|  |  | **Cluster 1 (n=37)** | **Cluster 4**  **(n=38)** | **Cluster 7**  **(n=32)** | **Cluster 2**  **(n=22)** | **Cluster 6 (n=43)** | **Cluster 3 (n=16)** | **Cluster 5 (n=12)** |
| **Age, mean (SD)** | 47.4 (12.7) | 50.9 (11.6) | 52.4 (11.9) | 54.6 (13.0) | 38.7 (10.9) | 40.8 (10.3) | 48.6 (11.0) | 39.9 (9.1) |
| **Age groups**  20-29  30-39  40-49  50-59  60-80 | 21 (10.5)  40 (20.0)  51 (25.5)  52 (26.0)  36 (18.0) | 0 (0.0)  7 (18.9)  9 (24.4)  14 (37.8)  7 (18.9) | 3 (7.9)  3 (7.9)  7 (18.4)  14 (36.8)  11 (29.0) | 2 (6.3)  3 (9.4)  6 (18.7)  8 (25.0)  13 (40.6) | 6 (27.3)  9 (40.9)  3 (13.6)  3 (13.6)  1 (4.6) | 7 (16.3)  14 (32.6)  14 (32.6)  7 (16.2)  1 (2.3) | 1 (6.2)  2 (12.5)  5 (31.3)  5 (31.3)  3 (18.7) | 2 (16.7)  2 (16.7)  7 (58.3)  1 (8.3)  0 (0.0) |
| **Sex**  Male  Female | 145 (72.5)  55 (27.5) | 28 (75.7)  9 (24.3) | 26 (68.4)  12 (31.6) | 24 (75.0)  8 (25.0) | 17 (77.3)  5 (22.7) | 30 (69.8)  13 (39.2) | 11 (68.8)  5 (31.2) | 9 (75.0)  3 (25.0) |
| **Housing status**  Rented/owned accommodation  Drug rehabilitation institution  Low-threshold institution  Prison  Homeless/on the street | 124 (62.0)  10 (5.0)  28 (14.0)  1 (0.5)  37 (18.5) | 24 (64.9)  3 (8.1)  1 (2.7)  1 (2.7)  8 (21.6) | 22 (57.9)  5 (13.2)  4 (10.5)  0 (0.0)  7 (18.4) | 25 (78.1)  0 (0.0)  4 (12.5)  0 (0.0)  3 (9.4) | 16 (72.7)  0 (0.0)  2 (9.1)  0 (0.0)  4 (18.2) | 22 (51.1)  2 (4.7)  10 (23.3)  0 (0.0)  9 (20.9) | 11 (68.7)  0 (0.0)  2 (12.6)  0 (0.0)  3 (18.7) | 4 (33.3)  0 (0.0)  5 (41.7)  0 (0.0)  3 (25.0) |
| **Source of income***  Part- or full-time job  Welfare pension  Social  Other | 26 (13.1)  116 (58.6)  52 (26.3)  4 (2.0) | 6 (16.2)  22 (59.4)  9 (24.4)  0 (0.0) | 3 (7.9)  30 (79.0)  5 (13.1)  0 (0.0) | 5 (16.2)  17 (54.8)  8 (25.8)  1 (3.2) | 1 (4.6)  16 (72.7)  5 (22.7)  0 (0.0) | 10 (23.3)  11 (25.6)  22 (51.1)  0 (0.0) | 1 (6.3)  14 (87.4)  1 (6.3)  0 (0.0) | 0 (0.0)  6 (54.6)  2 (18.2)  3 (27.2) |
| **History of injecting drug use**  Yes  No | 183 (91.5)  17 (8.5) | 34 (91.9)  3 (8.1) | 33 (86.8)  5 (13.2) | 28 (87.5)  4 (12.5) | 22 (100.0)  0 (0.0) | 41 (95.3)  2 (4.7) | 15 (93.8)  1 (6.2) | 10 (83.3)  2 (16.7) |
| **Recent (past 3 months) injecting drug use**  Yes  No | 121 (60.5)  79 (39.5) | 20 (54.1)  17 (45.9) | 20 (52.6)  18 (47.4) | 12 (37.5)  20 (62.5) | 20 (90.9)  2 (9.1) | 32 (74.4)  11 (25.6) | 10 (62.5)  6 (37.5) | 7 (58.3)  5 (41.7) |
| **Recent sharing of injecting equipment**†  Yes  No  Unknown | 34 (28.1)  54 (44.6)  33 (27.3) | 3 (15.0)  13 (65.0)  4 (20.0) | 6 (30.0)  7 (35.0)  7 (35.0) | 5 (41.7)  6 (50.0)  1 (8.3) | 8 (40.0)  9 (45.0)  3 (15.0) | 5 (15.6)  11 (34.4)  16 (50.0) | 1 (10.0)  8 (80.0)  1 (10.0) | 6 (85.7)  0 (0.0)  1 (14.3) |
| **Preferred injected drug**‡  Heroin  Amphetamines  Other/mixed | 114 (64.8)  51 (29.0)  11 (6.3) | 21 (61.8)  13 (38.2)  0 (0.0) | 22 (78.6)  6 (21.4)  0 (0.0) | 16 (61.5)  9 (34.6)  1 (3.9) | 13 (59.0)  8 (36.4)  1 (4.6) | 30 (73.2)  4 (9.8)  7 (17.0) | 7 (46.7)  8 (53.3)  0 (0.0) | 5 (50.0)  3 (30.0)  2 (20.0) |
| **Current opioid agonist therapy**  Yes  No | 90 (45.0)  110 (55.0) | 13 (35.1)  24 (64.9) | 16 (42.1)  22 (57.9) | 14 (43.7)  18(56.3) | 11(50.0)  11(50.0) | 27(62.8)  16 (37.2) | 6(37.5)  10 (62.5) | 3 (25.0)  9 (75.0) |
| **Opioid agonist therapy drug**§  Methadone  Buprenorphine  Buprenorphine-naloxone  Other | 48 (53.3)  36 (40.0)  2 (2.2)  4 (4.4) | 6 (46.2)  6 (48.2)  0 (0.0)  1 (7.6) | 11 (68.8)  3 (18.7)  0 (0.0)  2 (12.5) | 10 (71.4)  4 (28.6)  0 (0.0)  0 (0.0) | 6 (54.5)  5 (45.5)  0 (0.0)  0 (0.0) | 9 (33.3)  15 (55.6)  2 (7.4)  1 (3.7) | 4 (66.7)  2 (33.3)  0 (0.0)  0 (0.0) | 2 (66.7)  1 (33.3)  0 (0.0)  0 (0.0) |
| **Stage of liver disease**¶  Mild or no liver fibrosis  Intermediate fibrosis  Compensated cirrhosis  Decompensated cirrhosis | 102 (52.0)  54 (27.6)  21 (10.7)  19 (9.7) | 15 (41.7)  12 (33.3)  6 (16.7)  3 (8.3) | 16 (43.2)  12 (32.4)  4 (10.8)  5 (13.6) | 10 (32.3)  9 (29.0)  3 (9.7)  9 (29.0) | 15 (68.2)  5 (22.8)  1 (4.5)  1 (4.5) | 25 (59.5)  12 (28.6)  4 (9.5)  1 (2.4) | 11 (68.8)  2 (12.5)  3 (18.7)  0 (0.0) | 10 (83.3)  2 (16.7)  0 (0.0)  0 (0.0) |
| **FIB-4 index, mean (SD)**** | 2.72 (5.95) | 4.5 (12.2) | 3.4 (4.3) | 3.9 (4.0) | 1.4 (1.3) | 1.5 (1.4) | 1.4 (0.9) | 0.9 (0.5) |
| **Hepatocellular carcinoma**  Yes  No or not assessed | 9 (4.5)  191 (95.5) | 3 (8.1)  34 (91.9) | 3 (7.9)  35 (92.1) | 2 (6.3)  30 (93.7) | 1 (4.5)  21 (95.5) | 0 (0.0)  43 (100.0) | 0 (0.0)  16 (100.0) | 0 (0.0)  12 (100.0) |
| **Renal function**  eGFR >60 ml/min/1.73 m^2^  eGFR 30-59 ml/min/1.73 m^2^  eGFR < 30 ml/min/1.73 m^2^ | 188 (94.0)  8 (4.0)  4 (2.0) | 34 (91.9)  2 (5.4)  1 (2.7) | 33 (86.8)  3 (7.9)  2 (5.3) | 29 (90.6)  2 (6.3)  1 (3.1) | 22 (100.0)  0 (0.0)  0 (0.0) | 43 (100.0)  0 (0.0)  0 (0.0) | 16 (100.0)  0 (0.0)  0 (0.0) | 11 (91.7)  1 (8.3)  0 (0.0) |
| **HIV co-infection**  Yes  No  Not assessed | 6 (3.0)  169 (84.5)  25 (12.5) | 1 (2.7)  36 (97.3)  0 (0.0) | 2 (5.3)  32 (84.2)  4 (10.5) | 1 (3.1)  17 (53.2)  14 (43.7) | 0 (0.0)  22(100.0)  0 (0.0) | 1 (2.3)  40 (93.0)  2 (4.7) | 0 (0.0)  16 (100.0)  0 (0.0) | 1 (8.3)  6 (50.0)  5 (41.7) |
| **HBV co-infection (HBsAg+)**  Yes  No  Not assessed | 1 (0.5)  180 (90.0)  19 (9.5) | 0 (0.0)  21 (100.0)  0 (0.0) | 0 (0.0)  37 (97.4)  1 (2.6) | 0 (0.0)  20 (62.5)  12 (37.5) | 0 (0.0)  21 (95.4)  1 (4.6) | 0 (0.0)  41 (95.4)  2 (4.6) | 1 (6.3)  15 (93.7)  0 (0.0) | 0 (0.0)  9 (75.0)  3 (25.0) |
| **HCV genotype**  Genotype 1  Genotype 2  Genotype 3  Genotype 4-6  Not genotyped | 47 (23.5)  7 (3.5)  48 (24.0)  6 (3.0)  92 (46.0) | 7 (18.9)  1 (2.7)  8 (21.6)  0 (0.0)  21 (56.8) | 11 (28.9)  2 (5.3)  8 (21.1)  2 (5.3)  15 (39.4) | 7 (21.9)  2 (6.3)  8 (25.0)  2 (6.3)  13 (40.5) | 5 (22.7)  1 (4.5)  3 (13.6)  0 (0.0)  13 (59.2) | 13 (30.2)  1 (2.3)  18 (41.9)  1 (2.3)  10 (23.3) | 3 (18.7)  0 (0.0)  1 (6.3)  0 (0.0)  12 (75.0) | 1 (8.3)  0 (0.0)  2 (16.7)  1 (8.3)  8 (66.7) |
| **Days of hospitalization, median (IQR)** | 6 (3-13) | 4 (1-7) | 3.5 (2-6) | 4.5 (2.5-9) | 8.5 (6-13) | 11 (5-15) | 14.5 (7-25.5) | 11 (4-46) |
| **Main discharge diagnosis**  Drug related  Infectious diseases  Gastroenterology/hepatology  Mental health  Cardiopulmonary  Alcohol related  Other | 93 (46.5)  33 (16.5)  24 (12.0)  15 (7.5)  14 (7.0)  7 (3.5)  14 (7.0) | 8 (21.6)  13 (35.2)  5 (13.5)  0 (0.0)  6 (16.2)  1 (2.7)  4 (10.8) | 8 (21.0)  12 (31.6)  7 (18.4)  0 (0.0)  5 (13.2)  2 (5.3)  4 (10.5) | 3 (9.4)  8 (25.0)  12 (37.5)  0 (0.0)  3 (9.4)  0 (0.0)  6 (18.7) | 21 (95.5)  0 (0.0)  0 (0.0)  0 (0.0)  0(0.0)  1 (4.5)  0 (0.0) | 41 (95.3)  0 (0.0)  0 (0.0)  0 (0.0)  0 (0.0)  2 (4.7)  0 (0.0) | 6 (37.5)  0 (0.0)  0 (0.0)  10 (62.5)  0 (0.0)  0 (0.0)  0 (0.0) | 6 (50.0)  0 (0.0)  0 (0.0)  5 (41.7)  0 (0.0)  1 (8.3)  0 (0.0) |
| **Charlson comorbidity index**  0-1  2-3  4-5  $\geq$6 | 101 (50.5)  43 (21.5)  33 (16.5)  23 (11.5) | 12 (32.5)  10 (27.0)  9 (24.3)  6 (16.2) | 11 (28.9)  11 (28.9)  6 (15.8)  10 (26.4) | 8 (25.0)  7 (21.9)  11 (34.4)  6 (18.7) | 18 (81.8)  3 (13.6)  0 (0.0)  1 (4.6) | 35 (81.4)  5 (11.6)  3 (7.0)  0 (0.0) | 7 (43.8)  6 (37.5)  3 (18.7)  0 (0.0) | 10 (83.4)  1 (8.3)  1 (8.3)  0 (0.0) |
| **Charlson comorbidity index, mean (SD)** | 2.6 (2.3) | 3.5 (2.7) | 3.7 (2.7) | 3.7 (2.2) | 1.5 (1.3) | 1.3 (0.8) | 2.2 (1.3) | 1.4 (0.9) |

**Supplementary** **table 4.** Baseline characteristics summarized by cluster and total. Numbers are shown as n (%) unless otherwise indicated. Missing values are excluded from percentages.

SD = standard deviation; IQR = interquartile range; FIB-4 = fibrosis-4; GFR = glomerular filtration rate; HIV = human immunodeficiency virus; HBV = hepatitis B virus

* Missing data for 2 participants (1 intervention, 1 control)

† Among those with recent (past 3 months) injecting drug use

‡ Among those with a history of injecting drug use; missing data for 7 participants (2 intervention, 5 control)

§ Among those with current opioid agonist therapy

¶ Based on liver stiffness measurements in 86, FIB-4 index in 107, and imaging in 3 participants; missing data for 4 control participants

**Among 193 participants with an available FIB-4 index

| **Diagnosis (ICD)** | **Total (n=200)** | **Intervention (n=98)** | **Control (n=102)** |
| --- | --- | --- | --- |
| **Drug related**  Intoxication (F11.0, F13.0, F15.0, F19.0)  Harmful drug use (F11.2, F13.2, F14.1, F14.2, F15.1, F15.2, F15.5, F19.1, F19.2, F19.4, F19.5, F19.7, T50.9) | 11  82 | 6  34 | 5  48 |
| **Infectious diseases**  Skin and soft tissue infection (A46.0, L03.1, L03.8, L02.4)  Pneumonia (J15.9, J21, J14, J18.9, J69.0)  Urinary tract infection (N10)  Osteomyelitis (M46.26)  Sepsis (A40.0)  HIV infection (B20.6)  Pyogenic arthritis/synovitis (M00.90, M68)  Myositis/rhabdomyolysis (M60.03, M62.9)  Covid-19 (U07.1)  Fever (R50.9) | 13  9  1  1  1  1  3  2  1  1 | 6  7  0  1  0  1  3  2  1  1 | 7  2  1  0  1  0  0  0  0  0 |
| **Gastroenterology/hepatology**  Esophageal varices (I85.0)  Hematemesis (K92.0)  Cirrhosis/liver failure (K65.9, K70.3, K74.6, K72.1, K72.0)  Hepatocellular carcinoma (C22.0)  Esophagitis (K20)  Other (R10.1, R41.8, E43, E86, K26.0)  Hepatitis C (B18.2) | 1  1  9  4  1  7  1 | 0  0  5  2  0  3  0 | 1  1  4  2  1  4  1 |
| **Mental health**  Paranoid schizophrenia (F20.0)  Acute psychosis (F22, F22.8, F23.3, F29, F31.2,)  Other (F31.2. F33.1, F60.3) | 7  5  3 | 5  1  2 | 2  4  1 |
| **Cardiopulmonary**  Heart failure (I50.0)  Hypertension (I10)  Chronic Obstructive Pulmonary Disorder (J44.1, J44.8)  Angina Pectoris (I20)  Other (R07.3, R07.4, C34.9, I33.9, I49.0, J86.9, L09.2) | 2  1  3  1  7 | 2  0  1  1  4 | 0  1  2  0  3 |
| **Alcohol related**  Various (F06.9, F10.0, F10.1, F10.2) | 7 | 5 | 2 |
| **Other**  Kidney failure (N10, N18.5)  Benign tumor not specified (D33)  Investigation for cancer (D41.4)  Various (D50.9, I61.0, Y4N, C80.9, D64.9, E11.9, L55, R63.4, S71.0. F05) | 2  1  2  9 | 1  0  0  4 | 1  1  2  5 |

**Supplementary** **table 5.** Main discharge diagnoses summarized by total and intervention condition.

ICD = International Classification of Diseases

|  | **Accomplished primary outcome (treatment completion within six months)** | | | **Accomplished secondary outcome (treatment initiation within six months)** | | |
| --- | --- | --- | --- | --- | --- | --- |
|  | **Total (n=103)** | **Intervention (n=67)** | **Control (n=36)** | **Total (n=131)** | **Intervention (n=84)** | **Control (n=47)** |
| Time to prescription, median days (IQR)  Time to treatment, median days (IQR) | 0 (0-22)  11 (2-40) | 0 (0-1)  4 (1-20) | 52 (5-78)  50 (15-93) | 1 (0-40)  14 (2-52) | 0 (0-1)  4.5 (1-22) | 51 (7-100)  71 (19-106) |
| **DAA regimen**  SOF/VEL  GLE/PIB  SOF/LDV  GZR/EBR  SOF/VEL/VOX | 57 (55.3)  27 (26.2)  17 (16.5)  2 (1.9)  0 (0) | 35 (52.2)  25 (37.3)  6 (9.0)  1 (1.5)  0 (0) | 22 (61.1)  2 (5.6)  11 (30.6)  1 (2.8)  0 (0) | 73 (55.7)  32 (24.4)  21 (16.0)  4 (3.1)  1 (0.8) | 43 (51.2)  30 (35.7)  7 (8.3)  3 (3.6)  1 (1.2) | 30 (63.8)  2 (4.3)  14 (29.8)  1 (2.1)  0 (0) |
| **Model of care**  Self-administered  Outpatient clinic  Low-threshold clinic  Institution/nursing home  Municipal nursing services | 40 (38.8)  28 (27.2)  17 (16.5)  14 (13.6)  4 (3.9) | 35 (52.2)  4 (6.0)  12 (17.9)  12 (17.9)  4 (6.0) | 5 (13.9)  24 (66.7)  5 (13.9)  2 (5.6)  0 (0) | 49 (37.4)  39 (29.8)  22 (16.8)  15 (11.5)  6 (4.6) | 44 (52.4)  6 (7.1)  16 (19.1)  12 (14.3)  6 (7.1) | 5 (10.6)  33 (70.1)  6 (12.8)  3 (6.4)  0 (0) |

**Supplementary** **table 6.** Treatment characteristics for participants who accomplished the primary and secondary outcomes summarized by total and intervention condition. Numbers are shown as n (%) unless otherwise indicated.

IQR = interquartile range; SOF/VEL = sofosbuvir/velpatasvir; GLE/PIB = glecaprevir/pibrentasvir; SOF/LDV = sofosbuvir/ledipasvir; GZR/EBR = grazoprevir/elbasvir; SOF/VEL/VOX = sofosbuvir/velpatasvir/voxilaprevir

|  | **Total (n=159)** | **Treatment completion (n=141)** | **Treatment discontinuation (n=18)** |
| --- | --- | --- | --- |
| **SVR**$\boldsymbol{\geq}$**12** | 104 (65.4) | 98 (69.5) | 6 (33.3) |
| **SVR 4-12** | 22 (13.8) | 22 (15.6) | 0 (0) |
| **ETR** | 5 (3.1) | 5 (3.6) | 0 (0) |
| **Virologic failure** | 8 (5.0) | 4 (2.8) | 4 (22.2) |
| **Missing data** | 20 (12.6) | 12 (8.5) | 8 (44.4) |

**Supplementary table 7.** Virologic outcomes among participants who initiated treatment within data lock, summarized by total, treatment completion (proxy for cure) and treatment discontinuation. Numbers are shown as n (%).

SVR = Sustained Virologic Response; ETR = End of Treatment Response

|  | **Total**  **(n=200)** | **Intervention (n=98)** | **Control**  **(n=102)** | **Initiated treatment (n=159)** | **Not initiated (n=41)** | **Achieved primary outcome (n=103)** | **Failed primary outcome (n=97)** |
| --- | --- | --- | --- | --- | --- | --- | --- |
| **Causes of death** |  |  |  |  |  |  |  |
| Liver failure $\pm$ hepatocellular carcinoma | 7 | 3 | 4 | 2 | 5 | 1 | 6 |
| End-stage renal disease | 6 | 4 | 2 | 6 | 0 | 4 | 2 |
| Suicide | 3 | 3 | 0 | 3 | 0 | 2 | 1 |
| Septicemia | 2 | 1 | 1 | 0 | 2 | 0 | 2 |
| Coronary heart disease | 2 | 1 | 1 | 0 | 2 | 0 | 2 |
| Pulmonary disease | 2 | 1 | 1 | 0 | 2 | 0 | 2 |
| Other malignancy | 1 | 0 | 1 | 0 | 1 | 0 | 1 |
| Unknown | 1 | 1 | 0 | 1 | 0 | 1 | 0 |
| *Total* | *24* | *14* | *10* | *12* | *12* | *8* | *16* |
| **Mortality** |  |  |  |  |  |  |  |
| PY at risk | 328.7 | 121.9 | 206.8 | 273.4 | 55.3 | 162.8 | 165.9 |
| Crude mortality rate (per 100 PY) | 7.3 | 11.5 | 4.8 | 4.4 | 21.7 | 4.9 | 9.6 |
| 95% CI | 4.7-10.9 | 6.3-19.2 | 2.3-8.9 | 2.3-7.7 | 11.2-37.9 | 2.1-9.7 | 5.5-15.7 |

**Supplementary** **table 8.** Post-hoc analysis showing causes of death and mortality rates summarized by total, intervention condition, treatment initiation, and accomplishment of the primary outcome.

PY = person-years; CI = confidence interval

**REFERENCES**

1. Midgard H, Finbraten AK, Malme KB, Berg-Pedersen RM, Tanum L, Olsen IC, et al. Opportunistic treatment of hepatitis C virus infection (OPPORTUNI-C): study protocol for a pragmatic stepped wedge cluster randomized trial of immediate versus outpatient treatment initiation among hospitalized people who inject drugs. Trials. 2020;21(1):524.

2. Skaland M, Finbråten AK, Nilsen TIL, Dalgard O, Midgard H**.** Hepatitis C virus testing uptake among hospitalized people who inject drugs: Opportunities to enhance the HCV care continuum. The 10th International Conference on Health and Hepatitis Care in Substance Users, Glasgow, Scotland. 2022.

3. Midgard H, Ulstein K, Backe O, Foshaug T, Sorli H, Vennesland K, et al. Hepatitis C treatment and reinfection surveillance among people who inject drugs in a low-threshold program in Oslo, Norway. Int J Drug Policy. 2021;96:103165.

4. Charlson ME, Carrozzino D, Guidi J, Patierno C**.** Charlson Comorbidity Index: A Critical Review of Clinimetric Properties. Psychother Psychosom. 2022;91(1):8-35.

5. Hemming K, Taljaard M, Forbes A**.** Analysis of cluster randomised stepped wedge trials with repeated cross-sectional samples. Trials. 2017;18(1):101.

6. Wang R, De Gruttola V**.** The use of permutation tests for the analysis of parallel and stepped-wedge cluster-randomized trials. Stat Med. 2017;36(18):2831-43.

7. Therneau TM, Grambsch PM**.** Modeling Survival Data: Extending the Cox Model. New York: Springer. . 2000.

8. Lin DY, Wei LJ**.** The robust inference for the Cox proportional hazards model. . Journal of the American Statistical Association 1989;84: 1074–1078.

9. STATA**.** The Stata Journal 14. Number 2, pp. 363–380 2014.
