# Supplementary figures and images for: Opportunistic Treatment of Hepatitis C Infection Among Hospitalized People Who Inject Drugs (OPPORTUNI-C): A Stepped Wedge Cluster Randomized Trial
Source: Clin Infect Dis. 2023 Nov 22;78(3):582–90. doi: 10.1093/cid/ciad711 (PMC10954343; doi:10.1093/cid/ciad711)

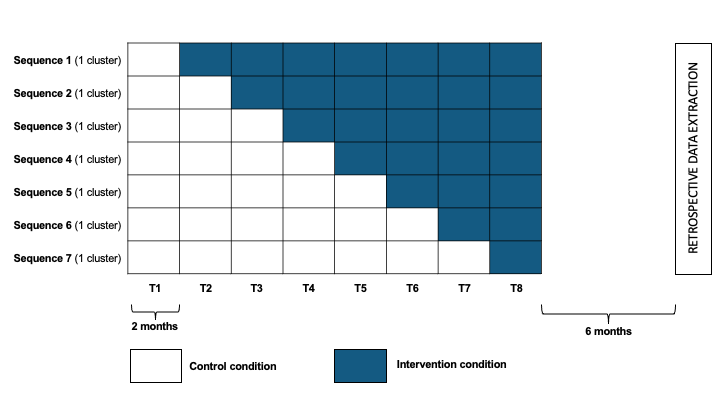

Supplement: ciad711_Supplementary_Data [file ciad711_supplementary_data.zip › Supplementary figure 1.tiff]
